# Convergent synthesis of the tetrasaccharide repeating unit of the cell wall lipopolysaccharide of *Escherichia coli* O40

**DOI:** 10.3762/bjoc.8.230

**Published:** 2012-11-22

**Authors:** Abhijit Sau, Anup Kumar Misra

**Affiliations:** 1 Bose Institute, Division of Molecular Medicine, P-1/12, C.I.T. Scheme VII-M, Kolkata-700054, India; FAX: +91-33-2355 3886

**Keywords:** *Escherichia coli*, glycosylation, lipopolysaccharide, O-antigen, tetrasaccharide

## Abstract

A tetrasaccharide repeating unit corresponding to the cell-wall lipopolysaccharide of *E. coli* O40 was synthesized by using a convergent block glycosylation strategy. A disaccharide donor was coupled to a disaccharide acceptor by a stereoselective glycosylation. A 2-aminoethyl linker was chosen as the anomeric protecting group at the reducing end of the tetrasaccharide. All glycosylation steps are significantly high yielding and stereoselective.

## Introduction

**Figure 1:**
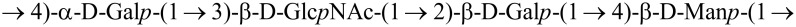
Structure of the tetrasaccharide repeating unit of the cell-wall lipopolysaccharide of *Escherichia coli* O40.

Infantile diarrhoea is one of the major causes of morbidity and mortality in infancy in developing countries [1]. Among several factors, *Escherichia coli* (*E. coli*) infection is one of the major causes of diarrhoeal disease in the developing countries [2]. *E. coli* are Gram-negative opportunistic pathogens and belong to the genus *Enterobacteriaceae*. In general, *E. coli* is considered as a friendly organism present in the normal intestinal flora of humans and animals and can kill harmful bacteria by producing vitamins and other immunostimulants [3]. However, a number of *E. coli* strains acquire virulence factors and cause severe intestinal and urinary-tract infections [4–5]. *E. coli* serotypes are generally classified based on the somatic, flagella and capsular antigens [6]. Diarrhoea-causing *E. coli* strains are broadly classified in four categories: (a) Enteropathogenic *E. coli* infects through the production of heat-labile and heat-stable toxins; (b) enteroinvasive *E. coli* acts through the invasion of the host body; (c) enteropathogenic *E. coli* infects by adhering to the membrane of the host intestine; and (d) verotoxin *E. coli* infects by the production of verotoxin or shiga toxin [7]. Recently, Zhao et al. reported the structure of the repeating unit of the cell-wall antigenic lipopolysaccharide of *E. coli* O40 [8], which contains two D-galactosyl moieties with alpha and beta linkage, one beta-linked D-glucosamine and one beta-linked D-mannosyl moiety (Figure 1).

Although several therapeutics have appeared in the past to control the diarrheal epidemics caused by *E. coli* infections, emergence of resistant strains is a serious concern in the development of therapeutics against this organism. Since, bacterial cell-wall lipopolysaccharides play important roles in the pathogenicity of the virulent strains, it would be pertinent to develop glycoconjugate therapeutics based on the cell-wall oligosaccharide haptens to reduce the number of infections [9–12]. In order to evaluate the therapeutic efficacy of the glycoconjugate derivatives it is essential to have a significant quantity of oligosaccharides, which is difficult to isolate from natural sources. Therefore, the development of a chemical synthetic strategy for the synthesis of the oligosaccharides and their close analogues can add momentum towards the preparation of glycoconjugate-based therapeutics. In this perspective, we report herein a concise chemical synthesis of the tetrasaccharide repeating unit of the cell-wall lipopolysaccharide of *E. coli* O40, using a convergent block synthetic strategy.

## Results and Discussion

**Figure 2:**
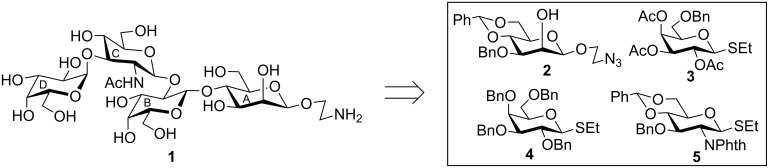
Structure of the synthesized tetrasaccharide **1** and its synthetic precursors.

The target tetrasaccharide **1** as its 2-aminoethyl glycoside was synthesized by a stereoselective glycosylation of a disaccharide acceptor **8** and a disaccharide thioglycoside donor **9** using a [2 + 2] block synthetic strategy. The disaccharide intermediates were synthesized from the suitably protected monosaccharide derivatives **2** [13], **3** [14], **4** [15] and **5** [16], which were prepared from the commercially available reducing sugars, by applying a series of functional group protection–deprotection methodologies (Figure 2). The synthetic strategy has a number of notable features, which include (a) stereoselective [2 + 2] block glycosylation; (b) application of general glycosylation reactions by using thioglycosides as glycosyl donors and a combination of *N*-iodosuccinimide (NIS) and perchloric acid supported over silica (HClO_4_–SiO_2_) [17–18] as glycosyl activator; (c) exploitation of the armed–disarmed glycosylation concept for the orthogonal activation of thioglycoside during the synthesis of disaccharide derivative **9** [19]; (d) use of aminoethyl linker as the anomeric protecting group; (e) removal of benzyl groups using a combination of triethylsilane and Pd(OH)_2_–C [20]; and (f) preparation of β-D-mannosidic moiety from the β-D-glucoside [13].

**Scheme 1:**
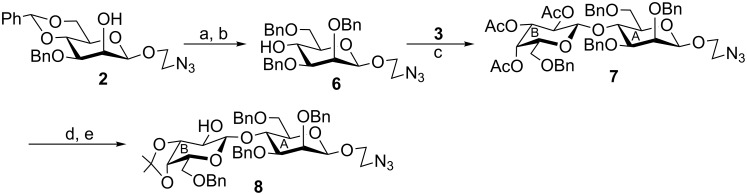
Synthesis of disaccharide derivative **8**. Reagents and conditions: (a) benzyl bromide, NaOH, DMF, room temperature, 1 h; (b) Et_3_SiH, I_2_, CH_3_CN, 0 °C, 1 h, 82%; (c) NIS, HClO_4_–SiO_2_, MS 4Å, CH_2_Cl_2_, −25 °C, 1 h, 77%; (d) 0.1 M CH_3_ONa, CH_3_OH, room temperature, 3 h; (e) 2,2-dimethoxypropane, *p*-TsOH, DMF, room temperature, 5 h, 74%.

Benzylation of 2-azidoethyl 3-*O*-benzyl-4,6-*O*-benzylidene-β-D-mannopyranoside (**2**) [13] (prepared from D-glucose in nine steps) by using benzyl bromide and sodium hydroxide [21] followed by reductive ring opening of the 4,6-*O*-benzylidene acetal with triethylsilane and iodine [22] furnished compound **6** in 82% yield. Stereoselective glycosylation of compound **6** with thioglycoside derivative **3** in the presence of a combination of *N*-iodosuccinimide (NIS) and HClO_4_–SiO_2_ [17] gave disaccharide derivative **7** in a 77% yield. Formation of compound **7** was confirmed from its spectral analysis [signals at δ 4.73 (d, *J* = 8.0 Hz, H-1_B_), 4.41 (br s, H-1_A_) in the ^1^H NMR and at δ 101.6 (C-1_A_), 100.7 (C-1_B_) in the ^13^C NMR spectra respectively]. Saponification of compound **7** by using sodium methoxide followed by 3,4-*O*-isopropylidenation with 2,2-dimethoxypropane and *p*-toluenesulfonic acid [23] furnished disaccharide derivative **8** in 74% yield (Scheme 1).

**Scheme 2:**
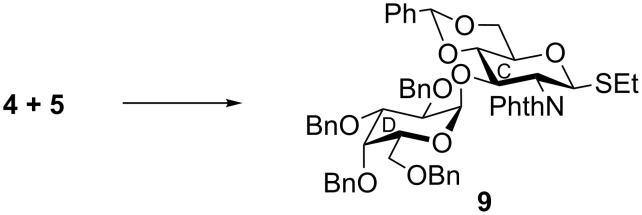
Synthesis of disaccharide derivative **9**. Reagents and conditions: (a) NIS, HClO_4_–SiO_2_, MS 4Å, CH_2_Cl_2_–Et_2_O, −25 °C, 1 h, 74%.

In a separate experiment, stereoselective glycosylation of thioglycoside derivative **4** with the thioglycoside acceptor **5** in the presence of a combination of NIS and HClO_4_–SiO_2_ [17] in dichloromethane–diethyl ether furnished disaccharide thioglycoside derivative **9** in a 74% yield together with a minor quantity of its other isomer (≈5%), which was separated by column chromatography. Formation of compound **9** was confirmed from its spectral analysis [δ 5.51 (d, *J* = 3.5 Hz, H-1_D_), 5.37 (d, *J* = 10.5 Hz, H-1_C_) in the ^1^H NMR and δ 97.4 (C-1_D_), 83.0 (C-1_C_) in the ^13^C NMR spectra, respectively]. During the synthesis of compound **9**, thioglycoside **4** acted as glycosyl donor and thioglycoside **5** acted as orthogonal glycosyl acceptor because of the difference in their reactivity following the “armed–disarmed glycosylation” concept [19,24] (Scheme 2).

**Scheme 3:**
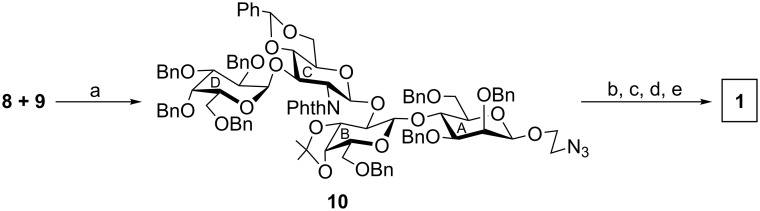
Synthesis of target tetrasaccharide **1**. Reagents and conditions: (a) NIS, HClO_4_–SiO_2_, CH_2_Cl_2_, −15 °C, 1 h, 71%; (b) NH_2_NH_2_·H_2_O, EtOH, 90 °C, 5 h; (c) acetic anhydride, pyridine, room temperature, 2 h; (d) 80% aq AcOH, 80 °C, 1.5 h; (e) triethylsilane, 20% Pd(OH)_2_–C, CH_3_OH, 6 h, room temperature, overall 60%.

Iodonium ion promoted [2 + 2] stereoselective glycosylation of compound **8** and compound **9** in the presence of NIS and HClO_4_–SiO_2_ [17] furnished tetrasaccharide derivative **10** in 71% yield. Formation of compound **10** was confirmed by its spectral analysis [signals at δ 101.6 (C-1_B_), 101.5 (PhC*H*), 100.8 (C-1_C_), 100.2 (C-1_A_), 97.3 (C-1_D_) in the ^13^C NMR spectrum]. Compound **10** was subjected to a sequence of reactions involving (a) removal of *N*-phthalimido group by using hydrazine hydrate [25]; (b) *N*-acetylation by using acetic anhydride and pyridine; (c) removal of isopropylidene ketal and benzylidene acetal by acid hydrolysis; and finally (d) removal of benzyl ethers by using triethylsilane and 20% Pd(OH)_2_–C [20] to furnish target compound **1**, which was purified through a Sephadex^®^ LH-20 column to give pure compound **1** in 60% overall yield. Spectral data of compound **1** confirmed its formation [signals at δ 5.31 (d, *J* = 8.5 Hz, H-1_C_), 5.15 (d, *J* = 3.5 Hz, H-1_D_), 4.63 (br s, H-1_A_), 4.34 (d, *J* = 8.5 Hz, H-1_B_) in the ^1^H NMR and at δ 100.7 (C-1_B_), 100.6 (C-1_B_), 99.6 (2 C, C-1_A_, C-1_C_) in the ^13^C NMR] (Scheme 3).

## Conclusion

In summary, synthesis of a tetrasaccharide repeating unit corresponding to the cell-wall lipopolysaccharide of *E. coli* O40 was achieved by using a convergent [2 + 2] block synthetic strategy. The yields are excellent in all reactions. A general reaction condition was used in all glycosylation reactions. All intermediates and final compounds were characterized by their spectral analysis. The armed–disarmed glycosylation concept was applied for the synthesis of disaccharide derivative **9**. A 2-Aminoethyl linker was used as the anomeric protecting group.

## Experimental

**General methods:** All reactions were monitored by thin-layer chromatography over silica-gel-coated TLC plates. The spots on TLC were visualized by warming ceric sulfate (2% Ce(SO_4_)_2_ in 2 N H_2_SO_4_)-sprayed plates on a hot plate. Silica gel 230–400 mesh was used for column chromatography. ^1^H and ^13^C NMR spectra were recorded on Brucker Avance 500 MHz by using CDCl_3_ as solvent and TMS as internal reference, unless stated otherwise. Chemical shift values are expressed in δ ppm. MALDI-MS were recorded on a Bruker Daltronics mass spectrometer. Commercially available grades of organic solvents of adequate purity were used in all reactions. HClO_4_–SiO_2_ was prepared following the method reported in the literature [18].

**2-Azidoethyl 2,3,6-tri-*O*-benzyl-β-D-mannopyranoside (6)**: To a solution of compound **2** (2.0 g, 4.68 mmol) in dry DMF (10 mL) were added benzyl bromide (1.2 mL, 10.09 mmol) and powdered NaOH (750.0 mg, 18.75 mmol) and the reaction mixture was stirred at room temperature for 1 h. The reaction mixture was diluted with water (100 mL) and extracted with CH_2_Cl_2_ (100 mL). The organic layer was washed with H_2_O, dried (Na_2_SO_4_) and concentrated. The crude product was passed through a short pad of SiO_2_ by using hexane–EtOAc (5:1) as eluant to give the *O*-benzylated product (2.2 g, 91%). A solution of the *O*-benzylated product (2.2 g, 4.25 mmol) in dry CH_3_CN (20 mL) was cooled to 0 °C. To the cooled reaction mixture were added Et_3_SiH (1.4 mL, 8.76 mmol) and I_2_ (250.0 mg, 0.98 mmol), and the reaction mixture was stirred at the same temperature for 1 h. The reaction mixture was diluted with CH_2_Cl_2_ (100 mL) and the organic layer was successively washed with saturated NaHCO_3_ and H_2_O, and then dried (Na_2_SO_4_) and concentrated. The crude product was purified over SiO_2_ by using hexane–EtOAc (4:1) as eluant to give pure compound **6** (1.7 g, overall 82%). White solid; mp 89–90 °C; [α]_D_^25^ −97 (*c* 1.0, CHCl_3_); IR (KBr): 3293, 2845, 2110, 1497, 1454, 1365, 1310, 1119, 1065, 779, 659, 599 cm^−1^; ^1^H NMR (500 MHz, CDCl_3_) δ 7.43–7.23 (m, 15H, Ar-H), 4.98 (d, *J* = 12.5 Hz, 1H, PhC*H*_2_), 4.75 (d, *J* = 12.5 Hz, 1H, PhC*H*_2_), 4.61, 4.58 (2 d, *J* = 12.0 Hz, 2H, PhC*H*_2_), 4.48 (br s, 1H, H-1), 4.45 (d, *J* = 12.0 Hz, 1H, PhC*H*_2_), 4.32 (d, *J* = 12.0 Hz, 1H, PhC*H*_2_), 4.15–4.11 (m, 1H,-OC*H*_2_-), 3.96 (br s, 1H, H-2), 3.94 (t, *J* = 9.5 Hz each, 1H, H-4), 3.85 (dd, *J* = 10.5, 3.5 Hz, 1H, H-6_a_), 3.75 (dd, *J* = 10.5, 6.5 Hz, 1H, H-6_b_), 3.66–3.62 (m, 1H, OC*H*_2_-), 3.58–3.53 (m, 1H, C*H*_2_N_3_), 3.46–3.42 (m, 1H, H-5), 3.34–3.30 (m, 1H, C*H*_2_N_3_), 3.29 (dd, *J* = 10.0, 3.5 Hz, 1H, H-3); ^13^C NMR (125 MHz, CDCl_3_) δ 138.6–127.4 (Ar-C), 101.8 (C-1), 81.3 (C-3), 75.4 (C-5), 74.4 (Ph*C*H_2_), 73.7 (Ph*C*H_2_), 73.5 (C-5), 71.1 (Ph*C*H_2_), 70.7 (C-6), 68.6 (O*C*H_2_), 68.0 (C-2), 50.9 (*C*H_2_N_3_); ESI-MS: 542.2 [M + Na]^+^; Anal. calcd for C_29_H_33_N_3_O_6_: C, 67.04; H, 6.40; found: C, 66.90; H, 6.58.

**2-Azidoethyl *O*-(2,3,4-tri-*O*-acetyl-6-*O*-benzyl-β-D-galactopyranosyl)-(1→4)-2,3,6-tri-*O*-benzyl-β-D-mannopyranoside (7)**: To a solution of compound **3** (1.4 g, 3.18 mmol) and compound **6** (1.5 g, 2.88 mmol) in anhydrous CH_2_Cl_2_ (10 mL) was added MS 4Å (2.0 g), and the reaction mixture was stirred at room temperature for 30 min under argon. The reaction mixture was cooled to −25 °C, and *N*-iodosuccinimide (NIS; 0.8 g, 3.55 mmol) and HClO_4_–SiO_2_ (25.0 mg) were added to it. After being stirred at same temperature for 1 h the reaction mixture was filtered through a Celite^®^ bed and washed with CH_2_Cl_2_ (100 mL). The organic layer was successively washed with 5% Na_2_S_2_O_3_, saturated NaHCO_3_ and water, and then dried (Na_2_SO_4_) and concentrated under reduced pressure to give the crude product. The crude product was purified over SiO_2_ by using hexane–EtOAc (7:1) as eluant to give pure compound **7** (2.0 g, 77%). Yellow oil; [α]_D_^25^ −13 (*c* 1.0, CHCl_3_); IR (neat): 3087, 2956, 2153, 1605, 1487, 1345, 1254, 1183, 1142, 1045, 999, 774, 734, 647, 542 cm^−1^; ^1^H NMR (500 MHz, CDCl_3_) δ 7.35–7.16 (m, 20H, Ar-H), 5.33 (d, *J* = 3.0 Hz, 1H, H-4_B_), 5.06 (t, *J* = 8.0 Hz each, 1H, H-2_B_), 4.87 (d, *J* = 12.0 Hz, 1H, PhC*H*_2_), 4.84 (dd, *J* = 10.5, 3.5 Hz, 1H, H-3_B_), 4.73 (d, *J* = 8.0 Hz, 1H, H-1_B_), 4.72–4.71 (2 d, *J* = 12.0 Hz each, 2H, PhC*H*_2_), 4.59 (d, *J* = 12.0 Hz, 1H, PhC*H*_2_), 4.47, 4.45 (2 d, *J* = 12.0 Hz each, 2H, PhC*H*_2_), 4.41 (br s, 1H, H-1_A_), 4.39 (d, *J* = 12.0 Hz, 1H, PhC*H*_2_), 4.19 (d, *J* = 12.0 Hz, 1H, PhC*H*_2_), 4.14 (t, *J* = 9.0 Hz each, 1H, H-4_A_), 4.10–4.05 (m, 1H, OC*H*_2_), 3.92 (d, *J* = 3.0 Hz, 1H, H-2_A_), 3.76–3.70 (m, 2H, H-6_abA_), 3.65–3.60 (m, 1H, OC*H*_2_), 3.54–3.49 (m, 2H, H-5_B_, C*H*_2_N_3_), 3.47 (dd, *J* = 10.0, 3.0 Hz, 1H, H-3_A_), 3.42–3.38 (m, 1H, H-5_A_), 3.32–3.22 (m, 3H, H-6_abB_, C*H*_2_N_3_), 1.99, 1.95, 1.90 (3 s, 9H, 3 COC*H*_3_); ^13^C NMR (125 MHz, CDCl_3_) δ 169.9, 169.8, 169.4 (3 *C*OCH_3_), 138.5–126.8 (Ar-C), 101.6 (C-1_A_), 100.7 (C-1_B_), 80.6 (C-5_B_), 75.7 (C-5_A_), 74.7 (C-2_A_), 74.3 (2 C, C-4_A_, Ph*C*H_2_), 73.7 (Ph*C*H_2_), 73.3 (Ph*C*H_2_), 71.7 (C-5_B_), 71.5 (Ph*C*H_2_), 71.3 (C-3_B_), 70.0 (C-2_B_), 68.6 (C-6_A_), 68.5 (O*C*H_2_), 67.4 (C-4_B_), 66.9 (C-6_B_), 50.8 (*C*H_2_N_3_), 20.7, 20.6, 20.5 (CO*C*H_3_); MALDI-MS: 920.3 [M + Na]^+^; Anal. calcd for C_48_H_55_N_3_O_14_: C, 64.20; H, 6.17; found: C, 64.06; H, 6.35.

**2-Azidoethyl *O*-(6-*O*-benzyl-3,4-*O*-isopropylidene-β-D-galactopyranosyl)-(1→4)-2,3,6-tri-*O*-benzyl-β-D-mannopyranoside (8)**: A solution of compound **7** (1.8 g, 2.0 mmol) in 0.1 M CH_3_ONa (25 mL) was stirred at room temperature for 2 h. The reaction mixture was neutralized with Dowex 50W X8 (H^+^) resin, filtered and concentrated. To a solution of the de-*O*-acetylated product in dry DMF (10 mL) was added 2,2-dimethoxypropane (0.7 mL, 5.69 mmol) followed by *p*-TsOH (0.2 g) and the reaction mixture was stirred at room temperature for 5 h. The reaction was quenched with Et_3_N (1 mL), the solvents were removed under reduced pressure, and the crude reaction mixture was diluted with CH_2_Cl_2_ (100 mL). The organic layer was washed with saturated NaHCO_3_, dried (Na_2_SO_4_) and concentrated to give the crude product, which was purified over SiO_2_ by using hexane–EtOAc (2:1) as eluant to give pure compound **8** (1.2 g, 74%). Yellow oil; [α]_D_^25^ −21 (*c* 1.0, CHCl_3_); IR (neat): 3418, 3030, 2926, 2198, 1743, 1711, 1646, 1390, 1253, 1099, 1053, 864, 754, 667, 531 cm^−1^; ^1^H NMR (500 MHz, CDCl_3_) δ 7.30–7.14 (m, 20H, Ar-H), 4.84 (d, *J* = 12.5 Hz, 1H, PhC*H*_2_), 4.63–4.40 (m, 6H, PhC*H*_2_), 4.38 (d, *J* = 8.0 Hz, 1H, H-1_B_), 4.33 (br s, 1H, H-1_A_), 4.30 (d, *J* = 12.5 Hz, 1H, PhC*H*_2_), 4.22 (t, *J* = 9.5 Hz each, 1H, H-4_A_), 4.02–3.98 (m, 2H, H-2_A_, OC*H*_2_), 3.91 (dd, *J* = 10.0, 3.5 Hz, H-3_B_), 3.83 (dd, *J* = 12.0, 5.5 Hz, 1H, H-6_aB_), 3.81 (d, *J* = 2.0 Hz, 1H, H-4_B_), 3.76 (dd, *J* = 12.0, 2.0, Hz, 1H, H-6_bB_), 3.71 (br s, 1H, H-5_B_), 3.70–3.68 (m, 1H, OC*H*_2_), 3.62–3.58 (m, 1H, H-6_aA_), 3.56–3.51 (m, 1H, C*H*_2_N_3_), 3.49 (dd, *J* = 8.0 Hz each, 1H, H-2_B_), 3.47–3.43 (m, 1H, H-6_bA_), 3.42–3.37 (m, 2H, H-3_A_, H-5_A_), 3.25–3.19 (m, 1H, C*H*_2_N_3_), 1.42, 1.25 (2 s, 6H, 2 C(C*H*_3_)_3_); ^13^C NMR (125 MHz, CDCl_3_) δ 138.7–127.3 (Ar-C), 109.8 (*C*(CH_3_)_2_), 102.5 (C-1_B_), 101.9 (C-1_A_), 80.6 (C-2_B_), 78.9 (C-3_B_), 75.2 (C-5_A_), 74.3 (Ph*C*H_2_), 74.2 (C-3_A_), 74.0 (C-4_B_), 73.8 (C-2_A_), 73.5 (Ph*C*H_2_), 73.4 (2C, C-4_A_, Ph*C*H_2_), 72.4 (C-5_B_), 71.3 (Ph*C*H_2_), 69.4 (O*C*H_2_), 69.3 (C-6_B_), 68.5 (C-6_A_), 50.8 (*C*H_2_N_3_), 28.2, 26.4 (C(*C*H_3_)_2_); MALDI-MS: 834.3 [M + Na]^+^; Anal. calcd for C_45_H_53_N_3_O_11_: C, 66.57; H, 6.58; found: C, 66.42; H, 6.75.

**Ethyl *O*-(2,3,4,6-tetra-*O*-benzyl-α-D-galactopyranosyl)-(1→3)-4,6-*O*-benzylidene-2-deoxy-2-*N*-phthalimido-1-thio-β-D-glucopyranoside (9):** To a solution of compound **4** (1.4 g, 2.39 mmol) and compound **5** (1.0 g, 2.26 mmol) in anhydrous CH_2_Cl_2_–Et_2_O (10 mL; 1:1 v/v) was added MS 4Å (2.0 g), and reaction mixture was stirred at room temperature for 30 min under argon. The reaction mixture was cooled to −25 °C and NIS (550.0 mg, 2.44 mmol) and HClO_4_–SiO_2_ (15.0 mg) were added. After being stirred at same temperature for 1 h the reaction mixture was filtered through a Celite^®^ bed and washed with CH_2_Cl_2_ (100 mL). The organic layer was successively washed with 5% Na_2_S_2_O_3_, saturated NaHCO_3_ and water, and then dried (Na_2_SO_4_) and concentrated under reduced pressure to give the crude product. The crude product was purified over SiO_2_ by using hexane–EtOAc (7:1) as eluant to give pure compound **9** (1.6 g, 74%). White solid; mp 67–68 °C; [α]_D_^25^ +39 (*c* 1.0, CHCl_3_); IR (KBr): 3417, 3063, 2870, 1774, 1715, 1610, 1495, 1485, 1385, 1216, 1099, 1023, 914, 753, 719 cm^−1^; ^1^H NMR (500 MHz, CDCl_3_) δ 7.76–6.91 (m, 29H, Ar-H), 5.51 (d, *J* = 3.5 Hz, 1H, H-1_D_), 5.37 (d, *J* = 10.5 Hz, 1H, H-1_C_), 5.32 (s, 1H, PhC*H*), 4.84 (t, *J* = 9.5 Hz each, 1H, H-3_C_), 4.77–4.58 (3 d, *J* = 12.0 Hz each, 3H, PhC*H*_2_), 4.46 (t, *J* = 9.5 Hz each, 1H, H-2_C_), 4.44 (d, *J* = 11.5 Hz, 1H, PhC*H*_2_), 4.34 (d, *J* = 11.5 Hz, 1H, PhC*H*_2_), 4.29 (t, *J* = 9.5 Hz each, 1H, H-4_C_), 4.19 (d, *J* = 11.5 Hz, 1H, PhC*H*_2_), 3.86 (br s, 2H, PhC*H*_2_), 3.85 (br s, 1H, H-4_D_), 3.81 (dd, *J* = 10.5, 3.0 Hz, 1H, H-2_D_), 3.72–3.67 (m, 3H, H-3_D_, H-5_C_, H-6_aD_), 3.58 (br s, 1H, H-5_D_), 3.33–3.31 (m, 1H, H-6_bD_), 3.23–3.19 (m, 1H, H-6_aC_), 2.80–2.77 (m, 1H, H-6_bC_), 2.67–2.56 (m, 2H, SC*H*_2_CH_3_), 1.12 (t, *J* = 7.5 Hz each, 3H, SCH_2_C*H*_3_); ^13^C NMR (125 MHz, CDCl_3_) δ 168.1, 167.9 (Phth*C*O), 138.9–123.1 (Ar-C), 101.7 (Ph*C*H), 97.4 (C-1_D_), 83.0 (C-1_C_), 81.7 (C-4_D_), 78.1 (C-3_D_), 75.4 (C-2_D_), 74.8 (C-5_D_), 74.7 (Ph*C*H_2_), 73.3 (2 C, C-3_C_, Ph*C*H_2_), 72.8 (Ph*C*H_2_), 71.8 (Ph*C*H_2_), 70.1 (C-5_C_), 69.4 (C-4_C_), 68.8 (C-6_D_), 67.7 (C-6_C_), 54.2 (C-2_C_), 24.0 (S*C*H_2_CH_3_), 14.9 (SCH_2_*C*H_3_); MALDI-MS: 986.3 [M + Na]^+^; Anal. calcd for C_57_H_57_NO_11_S: C, 71.01; H, 5.96; found: C, 70.88; H, 6.13.

**2-Azidoethyl *O*-(2,3,4,6-tetra-*O*-benzyl-α-D-galactopyranosyl)-(1→3)-*O*-(4,6-*O*-benzylidene-2-deoxy-2-*N*-phthalimido-β-D-glucopyranosyl)-(1→2)-*O*-(6-*O*-benzyl-3,4-*O*-isopropylidene-β-D-galactopyranosyl)-(1→4)-2,3,6-tri-*O*-benzyl-β-D-mannopyranoside (10):** To a solution of compound **8** (1.0 g, 1.23 mmol) and compound **9** (1.3 g, 1.35 mmol) in anhydrous CH_2_Cl_2_ (10 mL) was added MS 4Å (2.0 g), and reaction mixture was stirred at room temperature for 30 min under argon. The reaction mixture was cooled to −25 °C and NIS (350.0 mg, 1.55 mmol) and HClO_4_–SiO_2_ (10.0 mg) were added to it. After being stirred at same temperature for 1 h the reaction mixture was filtered through a Celite^®^ bed and washed with CH_2_Cl_2_ (100 mL). The organic layer was successively washed with 5% Na_2_S_2_O_3_, saturated NaHCO_3_ and water, and then dried (Na_2_SO_4_) and concentrated under reduced pressure to give the crude product. The crude product was purified over SiO_2_ by using hexane–EtOAc (7:1) as eluant to give pure compound **10** (1.5 g, 71%). White solid; mp 65–66 °C; [α]_D_^25^ +34 (*c* 1.0, CHCl_3_); IR (KBr): 3423, 3063, 3030, 2871, 2105, 1776, 1744, 1715, 1497, 1454, 1389, 1239, 1102, 1060, 874, 737, 721, 697, 600, 530 cm^−1^; ^1^H NMR (500 MHz, CDCl_3_) δ 7.76–6.96 (m, 49H, Ar-H), 5.52 (d, *J* = 3.5 Hz, 1H, H-1_D_), 5.42 (d, *J* = 8.0 Hz, 1H, H-1_C_), 5.25 (s, 1H, PhC*H*), 4.86–4.45 (m, 13H, PhC*H*_2_), 4.41 (d, *J* = 9.5 Hz, 1H, H-1_B_), 4.37 (t, *J* = 8.0 Hz, 1H, H-2_C_), 4.29 (d, *J* = 11.5 Hz, 1H, PhC*H*_2_), 4.24–4.20 (m, 4H, H-1_A_, H-3_C_, H-4_A_, OC*H*_2_), 4.13–4.08 (m, 2H, H-2_A_, OC*H*_2_), 3.91 (br s, 2H, PhC*H*_2_), 3.89–3.85 (m, 3H, H-2_D_, H-3_B_, H-3_D_), 3.84–3.75 (m, 3H, H-2_B_, H-4_C_, H-4_D_), 3.70 (t, *J* = 10.5 Hz each, 1H, H-6_aB_), 3.64–3.60 (m, 4H, H-4_B_, H-5_B_, H-5_D_, H-6_bB_), 3.58–3.43 (m, 5H, H-3_A_, H-6_aA_, H-6_aC_, H-6_abD_), 3.42–3.36 (m, 4H, H-5_A_, H-6_bA_, H-6_bC_, C*H*_2_N_3_), 3.34–3.26 (m, 2H, H-5_C_, C*H*_2_N_3_), 1.27, 1.25 (2 s, 6H, 2 C*H*_3_); ^13^C NMR (125 MHz, CDCl_3_) δ 138.6–126.3 (Ar-C), 109.5 (*C*(CH_3_)_2_), 101.6 (C-1_B_), 101.5 (PhC*H*), 100.8 (C-1_C_), 100.2 (C-1_A_), 97.3 (C-1_D_), 82.9 (C-4_C_), 82.7 (C-5_A_), 80.1 (C-5c), 78.7 (C-3_A_), 78.1 (C-4_D_), 76.2 (C-2_D_), 75.5 (C-2_B_), 74.9 (C-3_B_), 74.8 (C-5_D_), 74.7 (Ph*C*H_2_), 74.6 (C-3_D_), 73.9 (Ph*C*H_2_) 73.5 (C-3_C_), 73.4 (2 C, 2 Ph*C*H_2_), 73.2 (Ph*C*H_2_), 72.8 (Ph*C*H_2_), 72.6 (C-2_A_), 72.0 (Ph*C*H_2_), 71.7 (2 C, C-4_B_, Ph*C*H_2_), 69.3 (C-4_A_), 68.9 (C-6_C_), 68.8 (O*C*H_2_), 68.7 (C-6_A_), 68.4 (O*C*H_2_), 67.7 (2 C, C-6_B_, C-6_D_), 65.4 (C-5_B_), 56.0 (C-2_C_), 50.8 (*C*H_2_N_3_), 27.6, 25.7 (C(*C*H_3_)_2_); MALDI-MS: 1735.7 [M + Na]^+^; Anal. calcd for C_100_H_104_N_4_O_22_: C, 70.08; H, 6.12; found: C, 69.94; H, 6.30.

**2-Aminoethyl (α-D-galactopyranosyl)-(1→3)-(2-acetamido-2-deoxy-β-D-glucopyranosyl)-(1→2)-(β-D-galactopyranosyl)-(1→4)-β-D-mannopyranoside (1):** To a solution of compound **10** (500.0 mg, 0.29 mmol) in EtOH (5 mL) was added NH_2_NH_2_·H_2_O (0.1 mL) and the reaction mixture was stirred at 90 °C for 5 h. The solvents were removed under reduced pressure, and a solution of the crude product in acetic anhydride–pyridine (2 mL, 1:1 v/v) was kept at room temperature for 2 h and then concentrated. A solution of the crude product in 80% aq AcOH (10 mL) was stirred at 80 °C for 1.5 h and then concentrated. To a solution of the crude product in CH_3_OH (5 mL) were added Et_3_SiH (1.5 mL, 9.39 mmol) and 20% Pd(OH)_2_–C (100.0 mg) and the reaction mixture was stirred at room temperature for 6 h. The reaction mixture was filtered through a Celite^®^ bed and washed with CH_3_OH–H_2_O (2:1). The solvents were removed under reduced pressure and the product was passed through a Sephadex^®^ LH-20 column by using CH_3_OH–H_2_O (3:1) as eluant to furnish pure compound **1** (135.0 mg, 60%). Glass; [α]_D_^25^ +29 (*c* 1.0, H_2_O); IR (KBr): 3436, 2948, 1619, 1369, 1162, 669 cm^−1^; ^1^H NMR (500 MHz, D_2_O) δ 5.31 (d, *J* = 8.5 Hz, 1H, H-1_C_), 5.15 (d, *J* = 3.5 Hz, 1H, H-1_D_), 4.63 (br s, 1H, H-1_A_), 4.48 (t, *J* = 10.5 Hz each, 1H, H-3_C_), 4.34 (d, *J* = 8.5 Hz, 1H, H-1_B_), 4.09 (t, *J* = 10.0 Hz each, 1H, H-2_C_), 4.05–3.92 (m, 4H, H-2_A_, H-4_D_, H-5_D_, OC*H*_2a_), 3.90–3.80 (m, 4H, H-2_B_, H-3_A_, H-6_aB_, OC*H*_2b_), 3.78–3.57 (m, 11H, H-3_D_, H-4_B_, H-4_C_, H-6_abA_, H-6_bB_, H-6_abC_, H-6_abD_), 3.55–3.47 (m, 2H, H-2_D_, H-3_B_), 3.45–3.40 (m, 2H, H-5_A_, H-5_B_), 3.35–3.33 (m, 1H, H-5_C_), 3.20–3.15 (m, 2H, C*H*_2_NH_2_), 2.06 (s, 3H, COC*H*_3_); ^13^C NMR (125 MHz, D_2_O) δ 171.5 (*C*OCH_3_), 100.7 (C-1_B_), 100.6 (C-1_B_), 99.6 (2C, C-1_A_, C-1_C_), 81.7 (C-3_B_), 79.0 (C-3_C_), 78.5 (C-4_D_), 77.0 (C-3_D_), 75.5 (C-4_A_), 75.1 (C-4_B_), 73.5 (C-2_D_), 73.3 (C-5_A_), 71.3 (C-2_A_), 70.7 (C-4_C_), 70.6 (C-3_A_), 69.8 (2 C, C-5_C_, C-5_D_), 68.3 (2C, C-2_B_, C-5_B_), 65.5 (O*C*H_2_), 60.6 (C-6_B_), 60.5 (C-6_C_), 60.2 (C-6_A_), 59.2 (C-6_D_), 55.7 (C-2_C_), 39.5 (*C*H_2_NH_2_), 23.1 (CO*C*H_3_); MALDI-MS: 799.2 [M + Na]^+^; Anal. calcd for C_28_H_48_N_4_O_21_: C, 43.30; H, 6.23; found: C, 43.14; H, 6.45.

## Supporting Information

File 1 1D and 2D NMR spectra of compounds **1** and **6–10**.
