# Supplementary material for: Convergent synthesis of the tetrasaccharide repeating unit of the cell wall lipopolysaccharide of Escherichia coli O40
Source: Beilstein J Org Chem. 2012 Nov 22;8:2053–9. doi: 10.3762/bjoc.8.230 (PMC3511039; doi:10.3762/bjoc.8.230)
Supplement: File 1 — 1D and 2D NMR spectra of compounds 1 and 6–10. [file Beilstein_J_Org_Chem-08-2053-s001.pdf]

Supporting Information

for

Convergent synthesis of the tetrasaccharide repeating unit of the cell wall lipopolysaccharide of *Escherichia coli* O40

Abhijit Sau and Anup Kumar Misra*

Address: Bose Institute, Division of Molecular Medicine, P-1/12, C.I.T. Scheme VII-M, Kolkata-700054, India; FAX: +91-33-2355 3886

Email: Anup Kumar Misra - akmisra69@gmail.com

* Corresponding author

1D and 2D NMR spectra of compounds 1 and 6–10

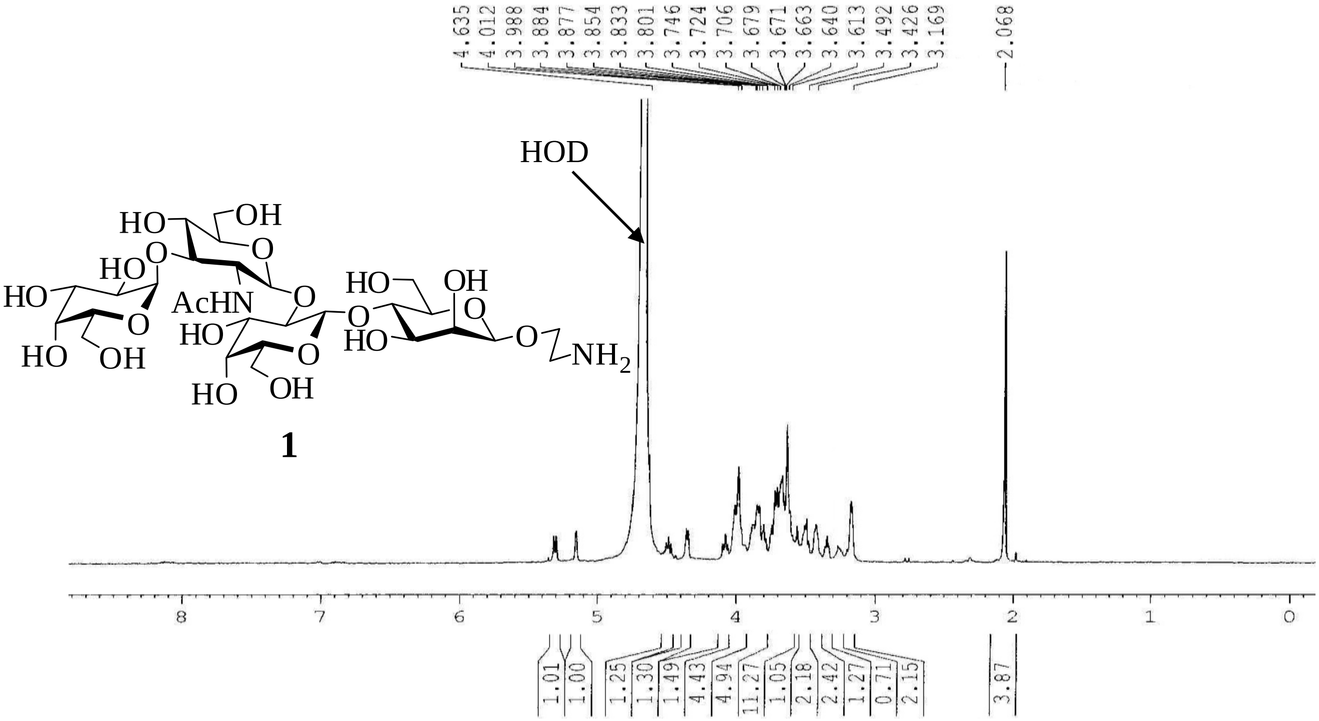
4.635
4.012
3.988
3.884
3.877
3.854
3.833
3.801
3.746
3.724
3.706
3.679
3.671
3.663
3.640
3.613
3.492
3.426
3.169
2.068
HOD
AcHN
NH2
1
8 7 6 5 4 3 2 1 0
1.01
1.00
1.25
1.30
1.49
4.43
4.94
11.27
1.05
2.18
2.42
1.27
0.71
2.15
3.87

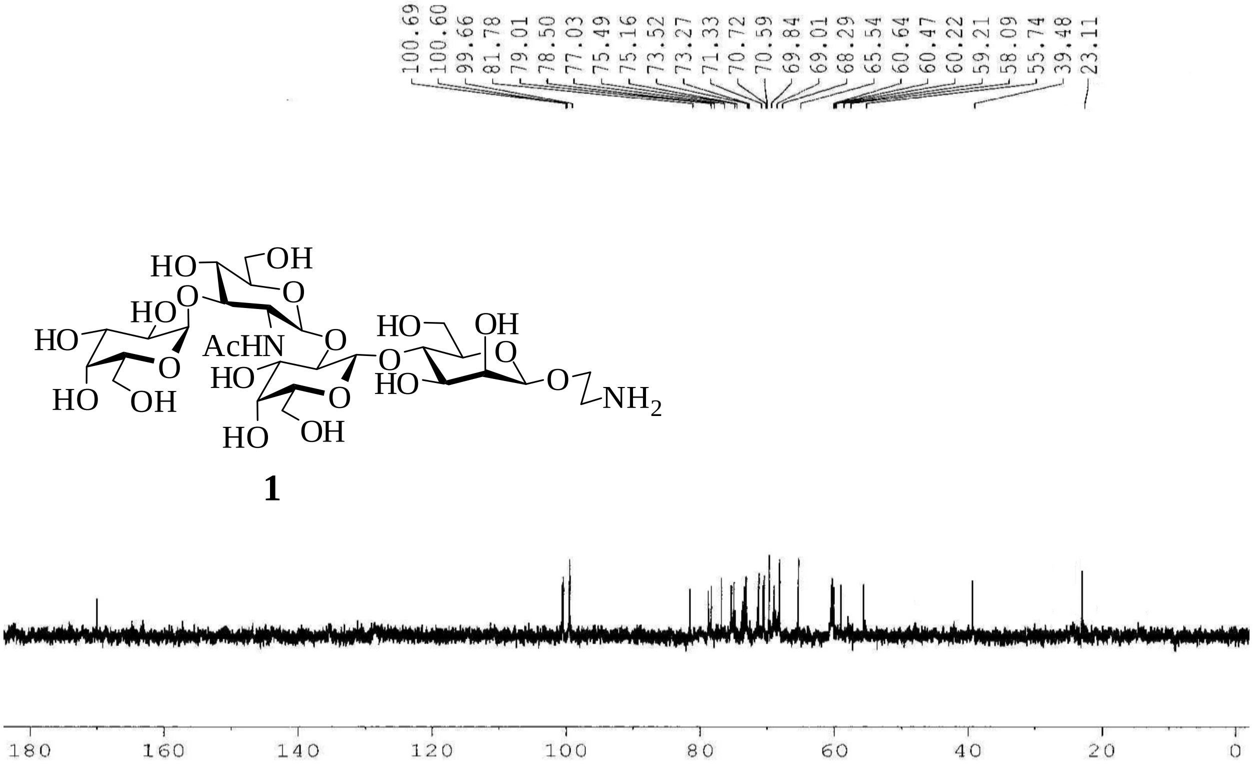
100.69
100.60
99.66
81.78
79.01
78.50
77.03
75.49
75.16
73.52
73.27
71.33
70.72
70.59
69.84
69.01
68.29
65.54
60.64
60.47
60.22
59.21
58.09
55.74
39.48
23.11
AcHN
NH2
1
180 160 140 120 100 80 60 40 20 0

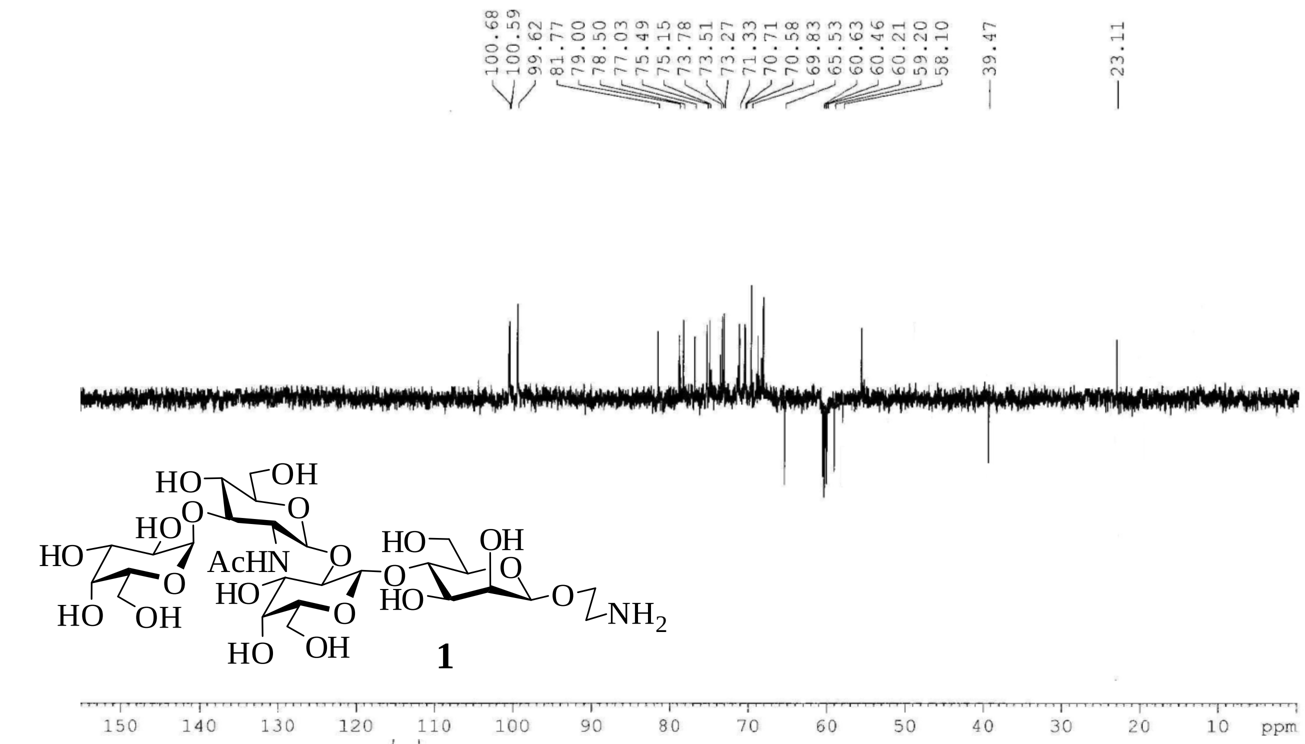
100.68
100.59
99.62
81.77
79.00
78.50
77.03
75.49
75.15
73.78
73.51
73.27
71.33
70.71
70.58
69.83
65.53
60.63
60.46
60.21
59.20
58.10
39.47
23.11
150 140 130 120 110 100 90 80 70 60 50 40 30 20 10 ppm
1

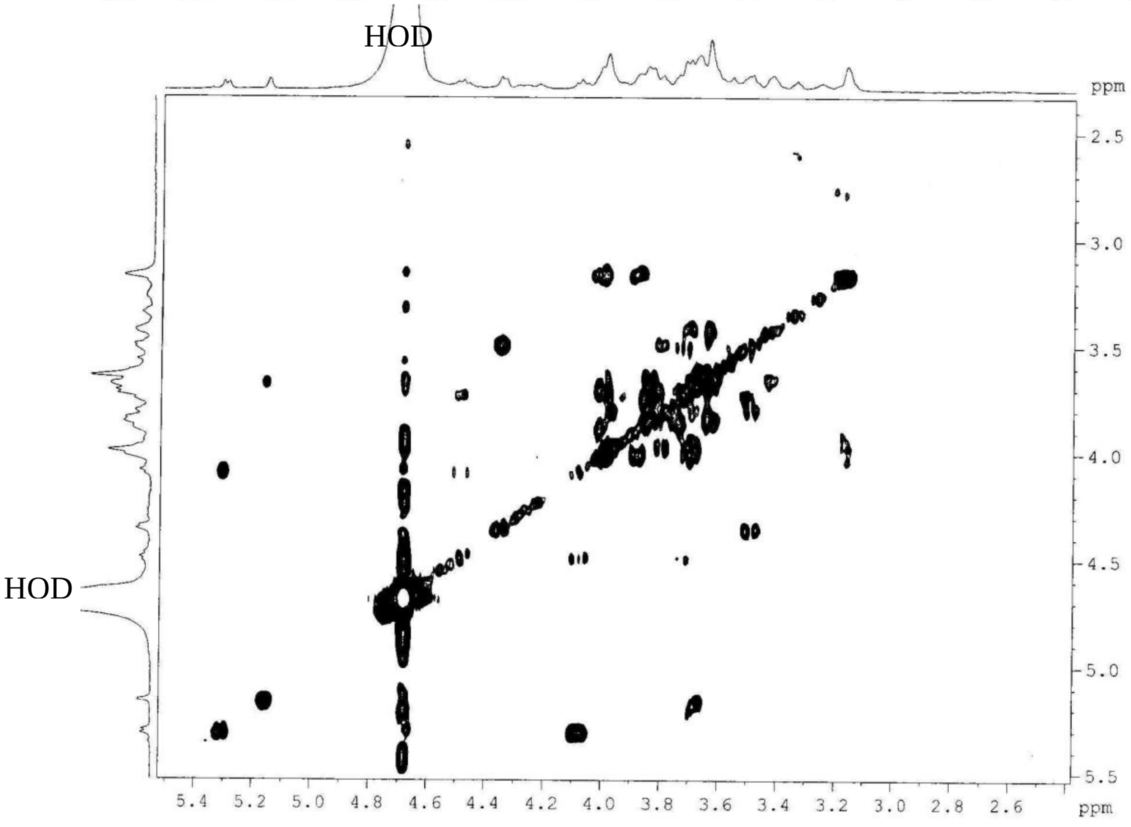
HOD
HOD
ppm
2.5
3.0
3.5
4.0
4.5
5.0
5.5
5.4 5.2 5.0 4.8 4.6 4.4 4.2 4.0 3.8 3.6 3.4 3.2 3.0 2.8 2.6
ppm

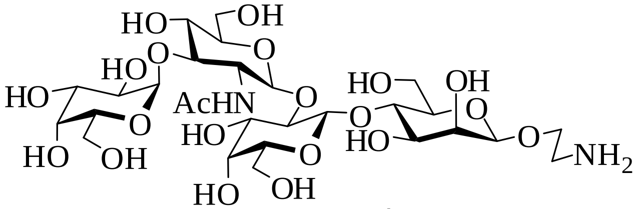

1

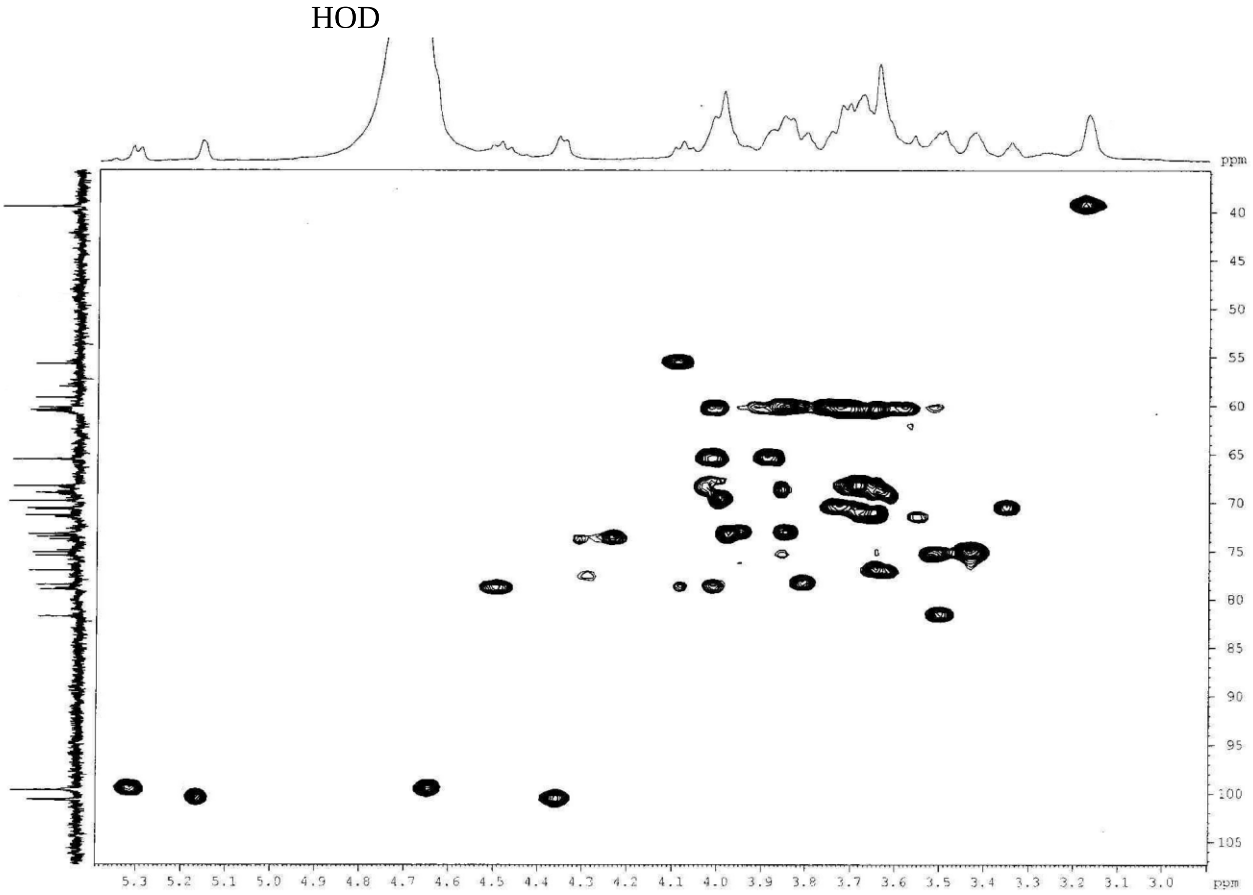
HOD
ppm
40
45
50
55
60
65
70
75
80
85
90
95
100
105
5.3 5.2 5.1 5.0 4.9 4.8 4.7 4.6 4.5 4.4 4.3 4.2 4.1 4.0 3.9 3.8 3.7 3.6 3.5 3.4 3.3 3.2 3.1 3.0
ppm

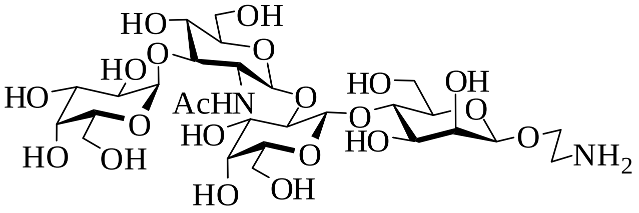
HO
OH
O
HO
O
HO
AcHN
O
HO
OH
O
HO
O
O
HO
HO
OH
HO
O
OH
O
NH2

1

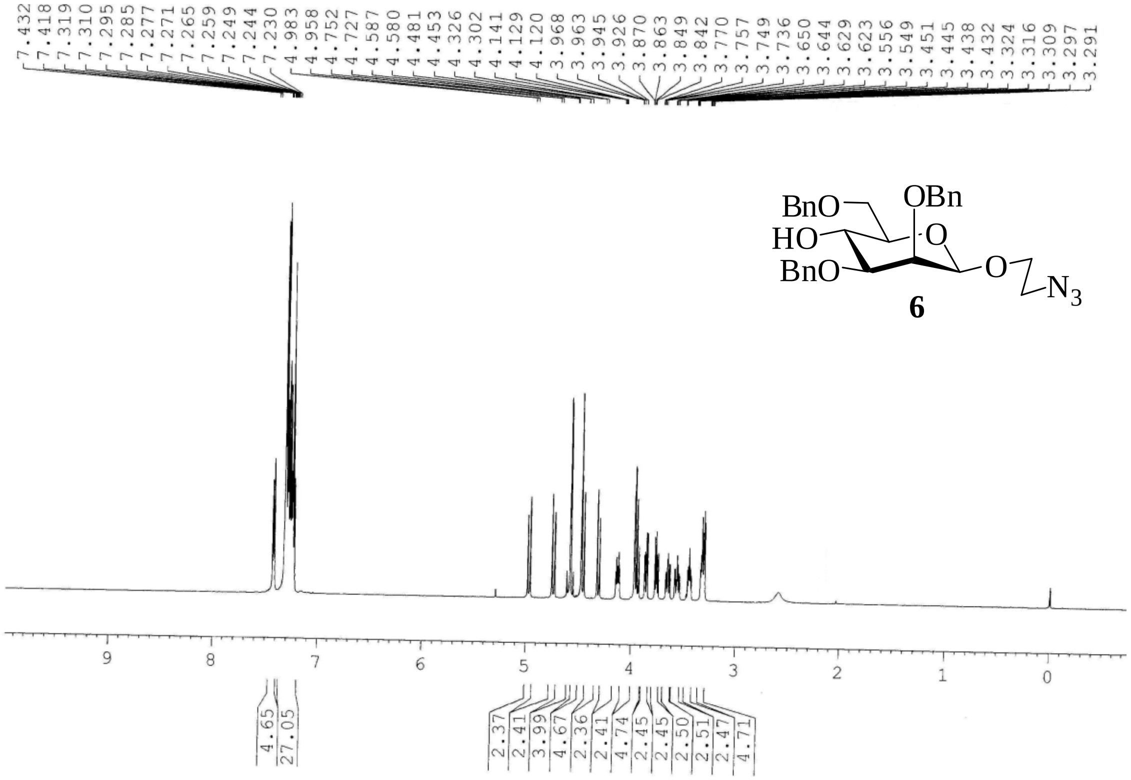
7.432
7.418
7.319
7.310
7.295
7.285
7.277
7.271
7.265
7.259
7.249
7.244
7.230
4.983
4.958
4.752
4.727
4.587
4.580
4.481
4.453
4.326
4.302
4.141
4.129
4.120
3.968
3.963
3.945
3.926
3.870
3.863
3.849
3.842
3.770
3.757
3.749
3.736
3.650
3.644
3.629
3.623
3.556
3.549
3.451
3.445
3.438
3.432
3.324
3.316
3.309
3.297
3.291
9
8
7
6
5
4
3
2
1
0
4.65
27.05
2.37
2.41
3.99
4.67
2.36
2.41
4.74
2.45
2.45
2.50
2.51
2.47
4.71
BnO
OBn
HO
O
BnO
O
N3

6

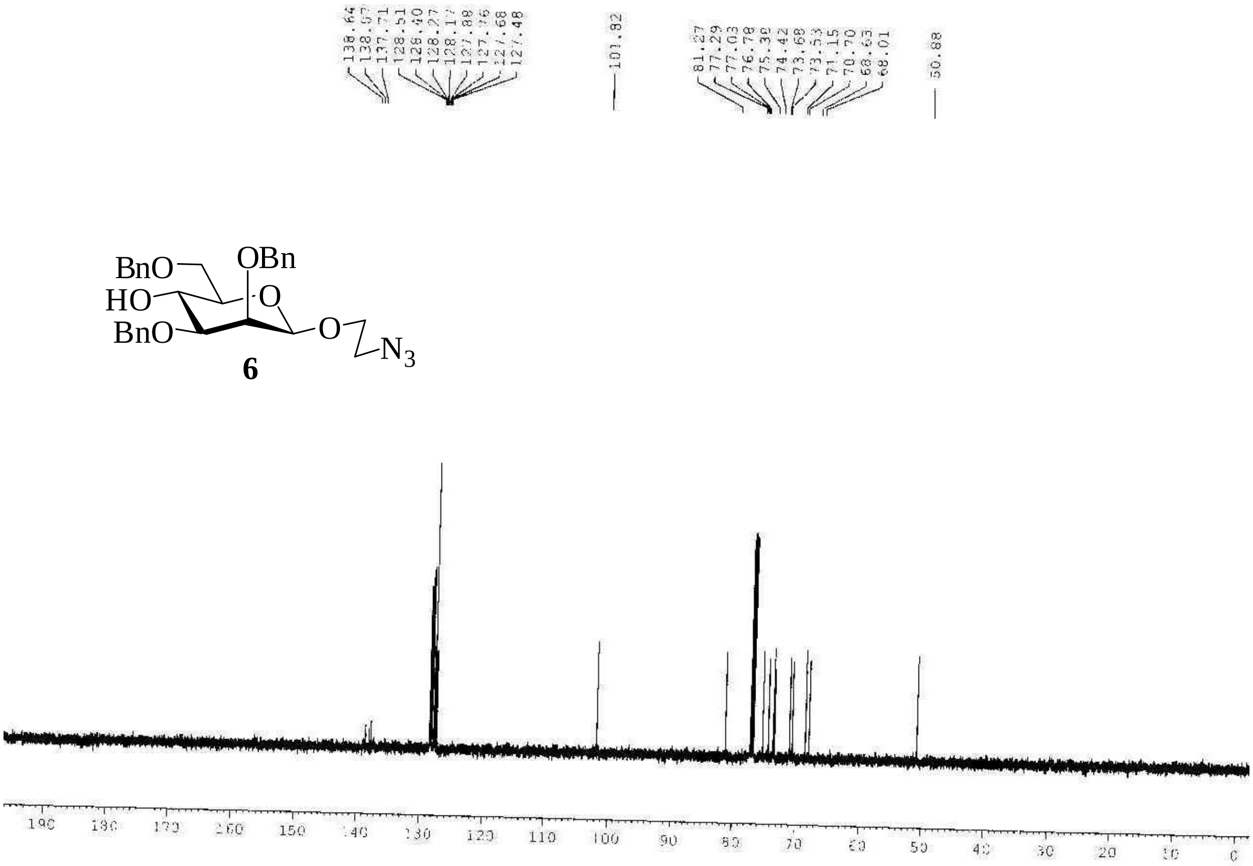
BnO
OBn
HO
O
BnO
O
N3
138.64
138.57
137.71
128.51
128.40
128.27
128.17
127.88
127.76
127.68
127.48
101.82
81.27
77.29
77.03
76.78
75.38
74.42
73.68
73.53
71.15
70.70
68.63
68.01
50.88
190
180
170
160
150
140
130
120
110
100
90
80
70
60
50
40
30
20
10
0

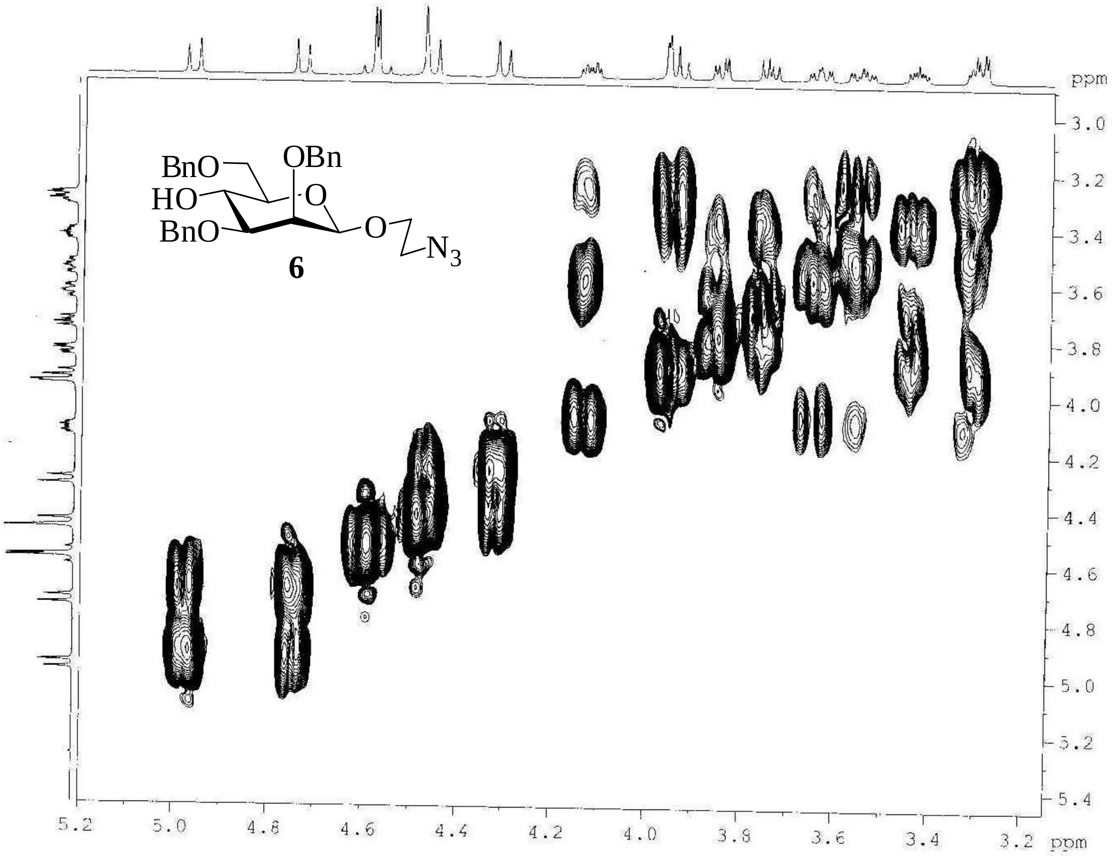
BnO
OBn
HO
O
BnO
O
N3
6
ppm
3.0
3.2
3.4
3.6
3.8
4.0
4.2
4.4
4.6
4.8
5.0
5.2
5.4
5.2
5.0
4.8
4.6
4.4
4.2
4.0
3.8
3.6
3.4
3.2
ppm

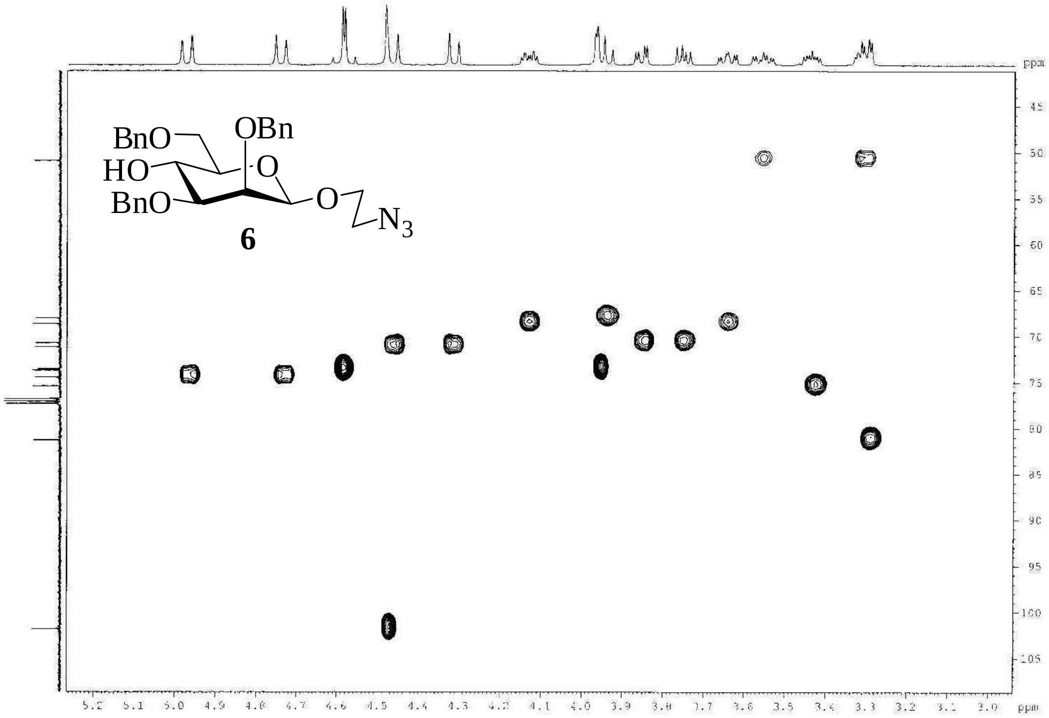
BnO
OBn
HO
O
BnO
O
N3
6
ppm

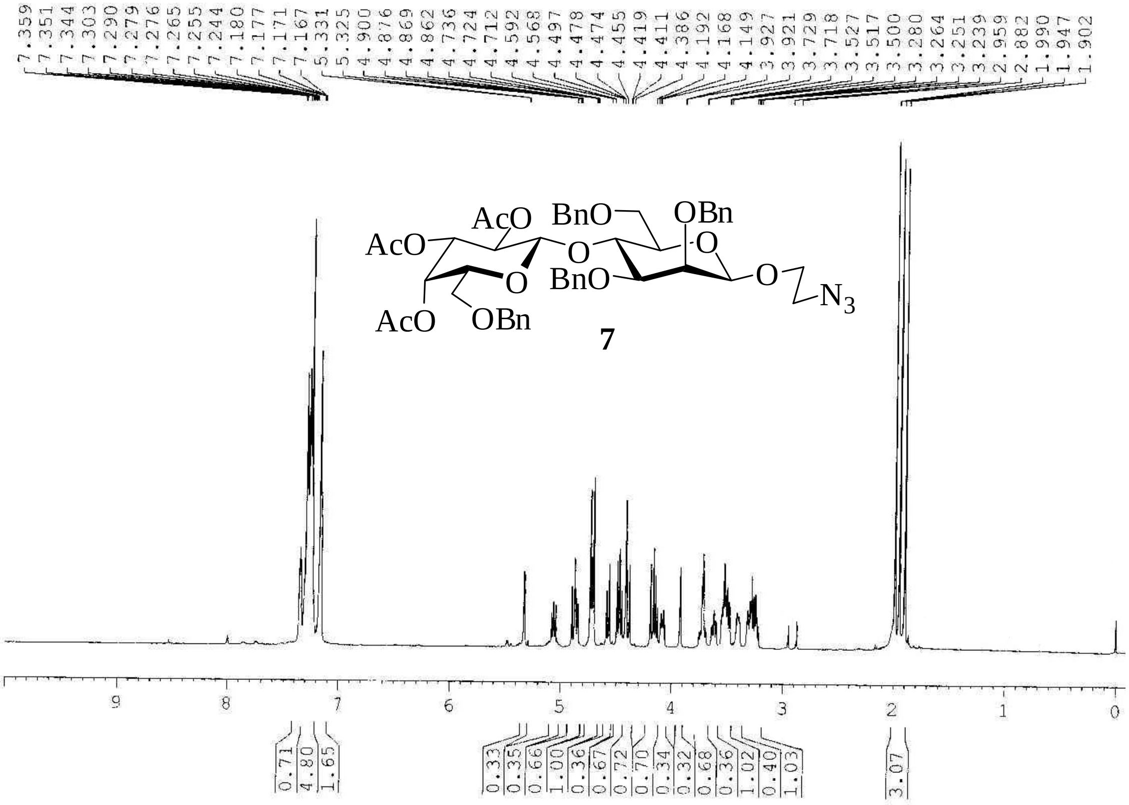
AcO
AcO
AcO
OBn
BnO
OBn
BnO
O
N3
7

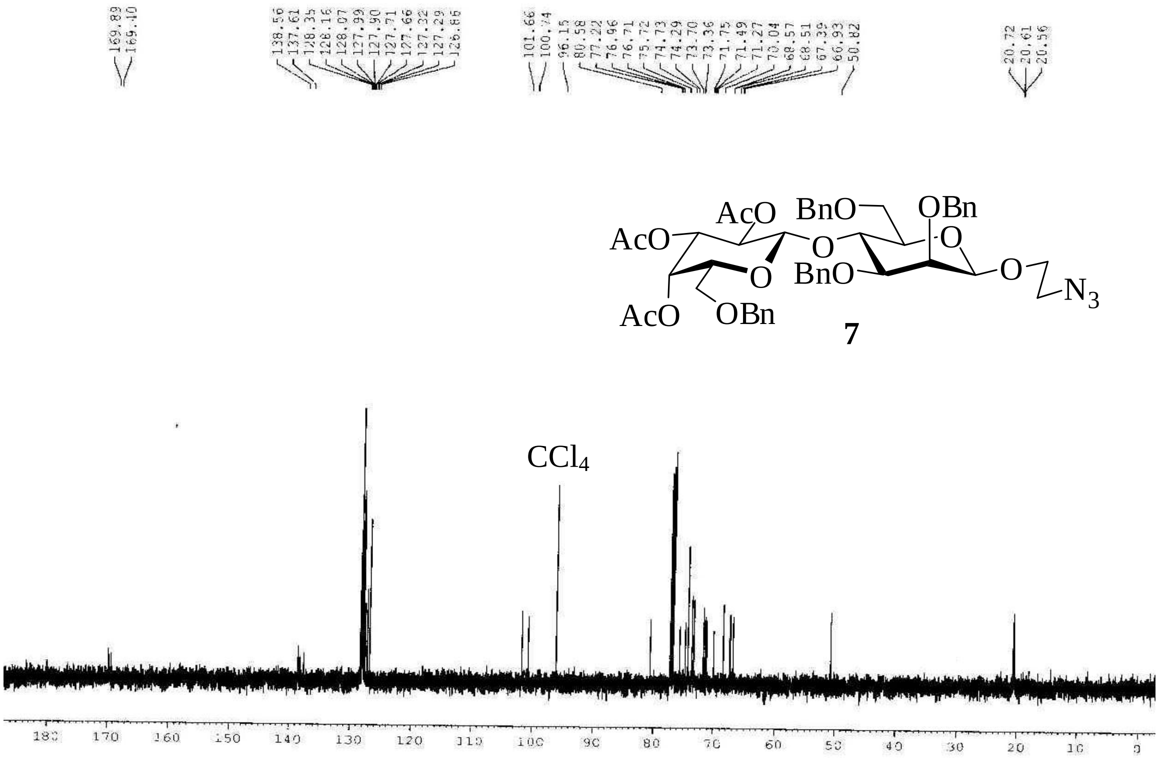
AcO
AcO
AcO
OBn
BnO
OBn
BnO
O
N3
7
CCl4

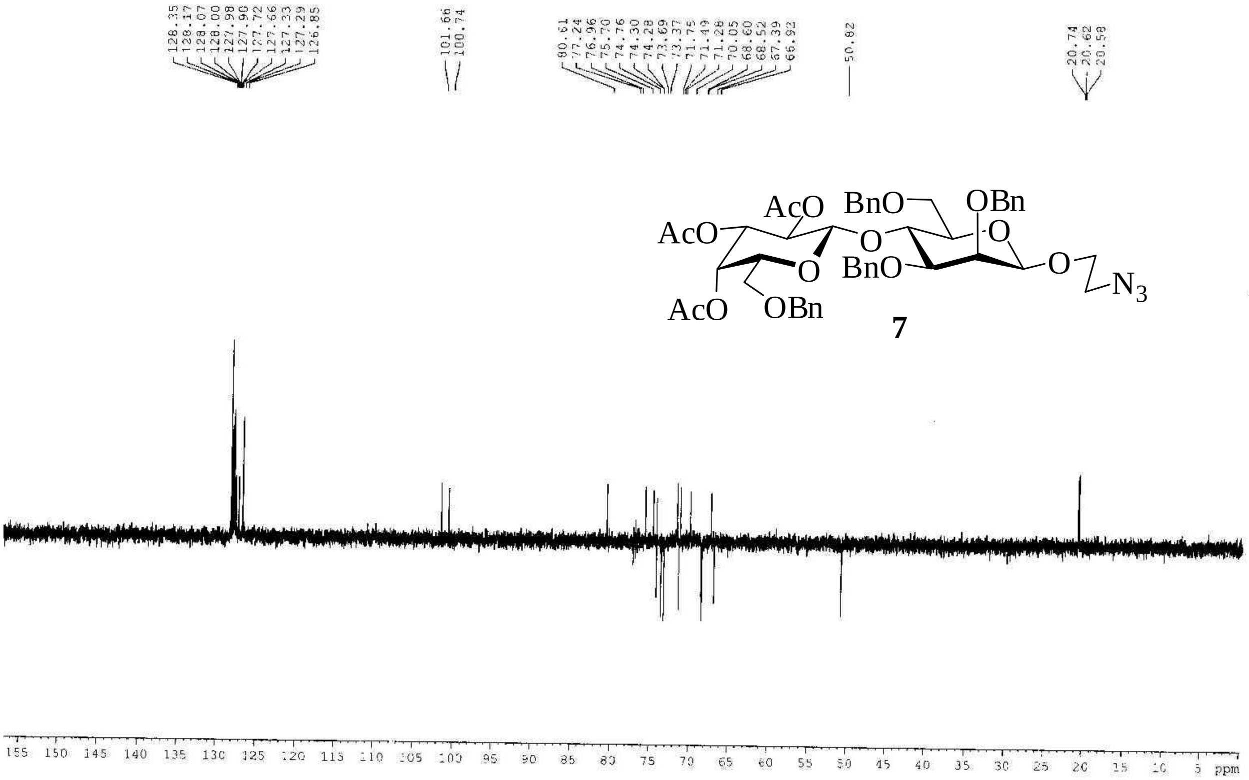
7
ppm

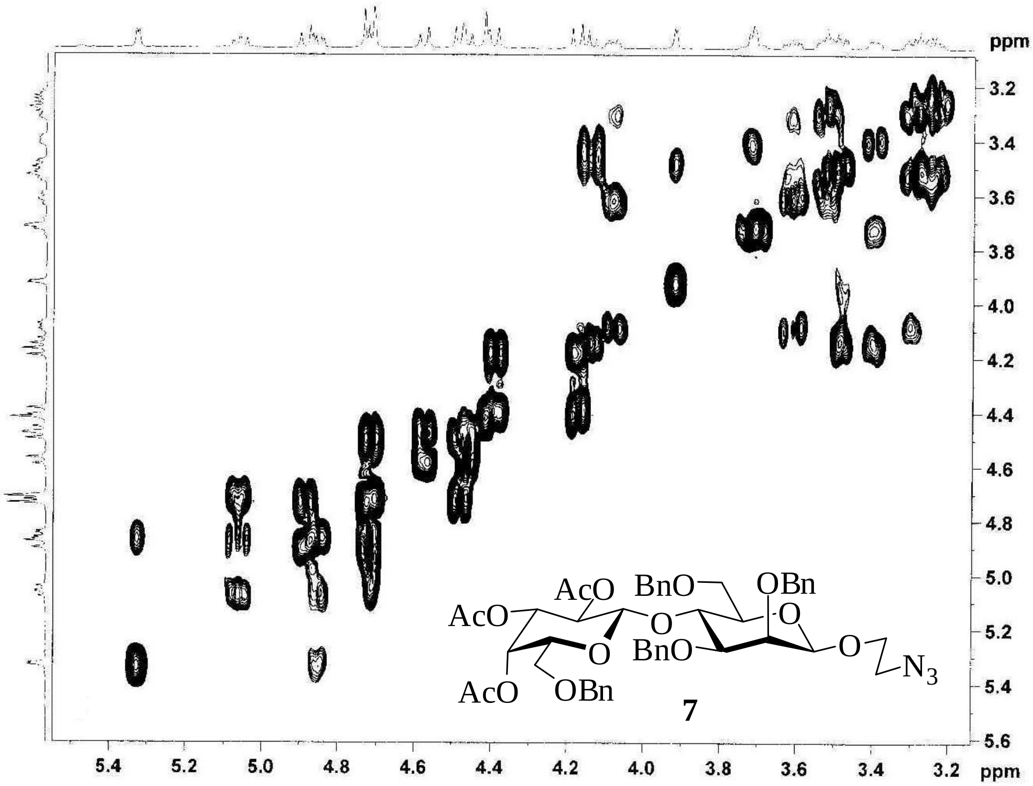
7
ppm

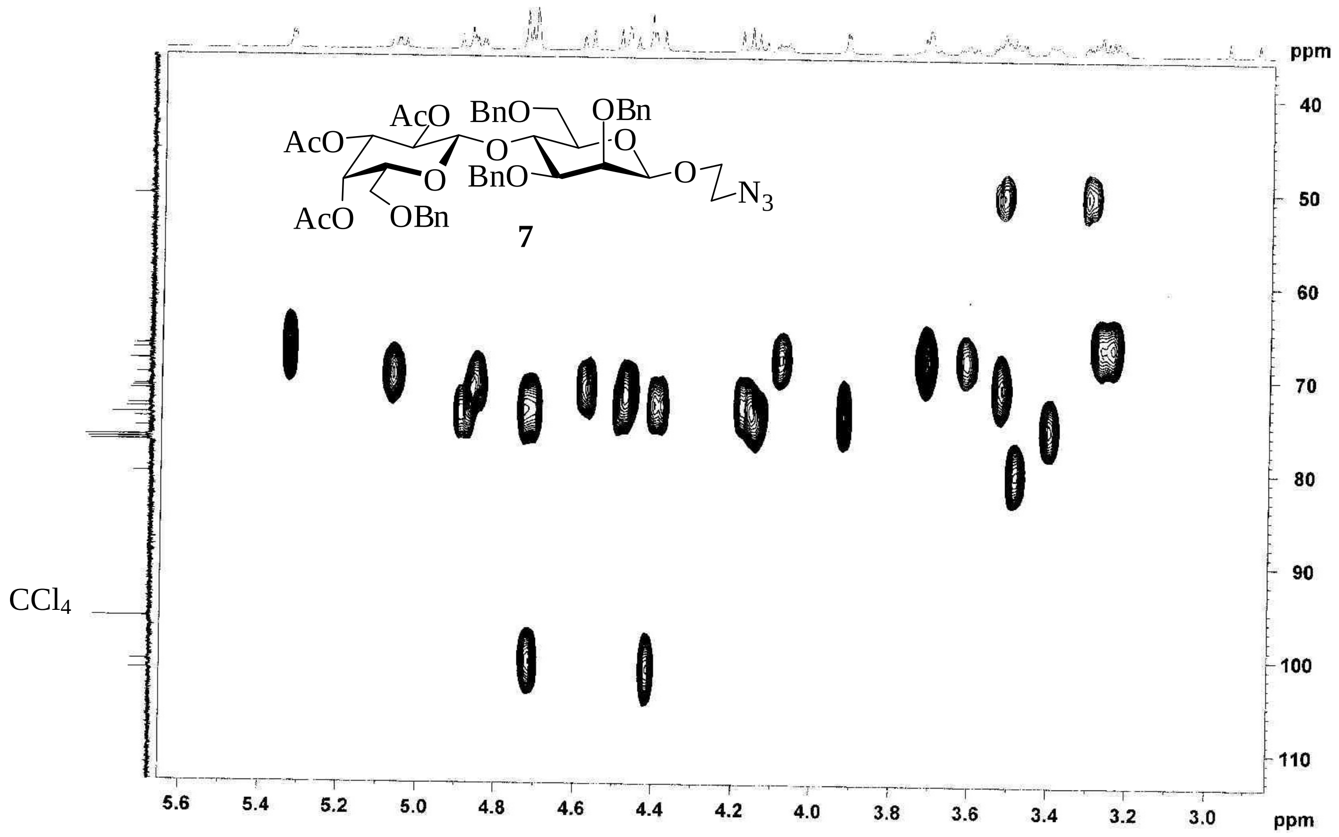
AcO
AcO
AcO
OBn
O
O
BnO
BnO
OBn
O
O
N3
7
ppm
40
50
60
70
80
90
100
110
5.6
5.4
5.2
5.0
4.8
4.6
4.4
4.2
4.0
3.8
3.6
3.4
3.2
3.0
ppm
CCl4

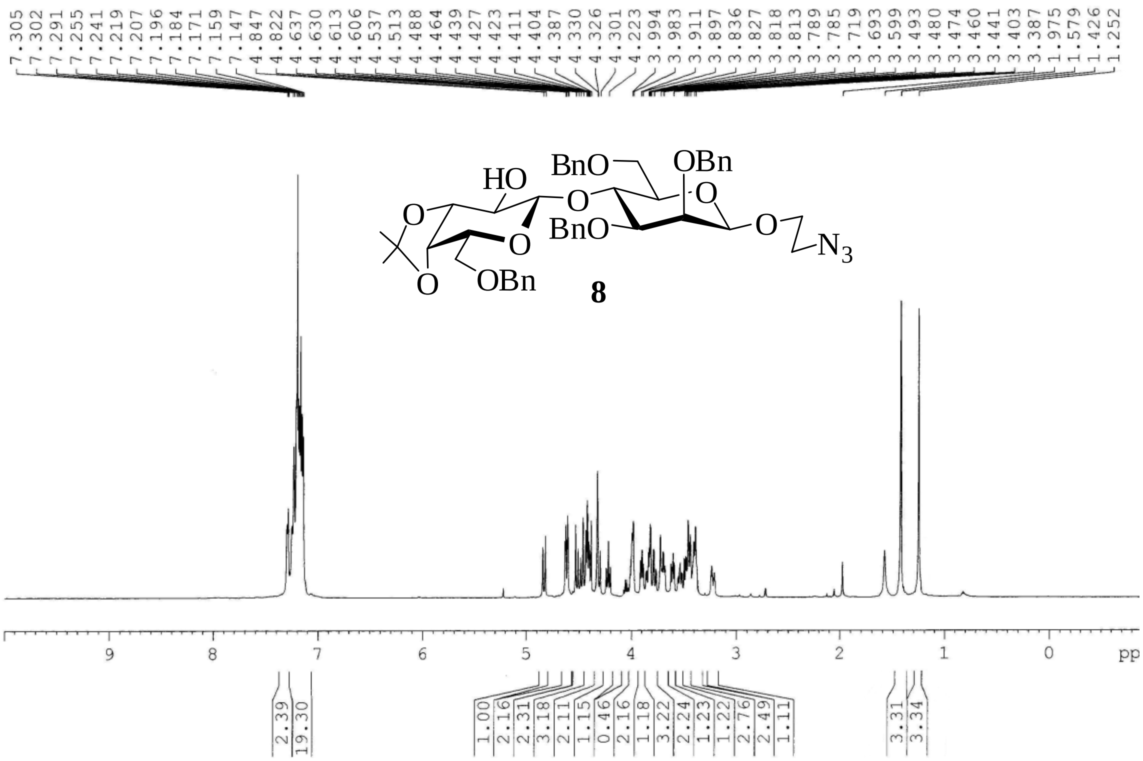
7.305 7.302 7.291 7.255 7.241 7.219 7.207 7.196 7.184 7.171 7.159 7.147 4.847 4.822 4.637 4.630 4.613 4.606 4.537 4.513 4.488 4.464 4.439 4.427 4.423 4.411 4.404 4.387 4.330 4.326 4.301 4.223 3.994 3.983 3.911 3.897 3.836 3.827 3.818 3.813 3.789 3.785 3.719 3.693 3.599 3.493 3.480 3.474 3.460 3.441 3.403 3.387 1.975 1.579 1.426 1.252
HO
BnO
OBn
O
BnO
O
N_3
O
OBn
8
9 8 7 6 5 4 3 2 1 0 pp
2.39 19.30 1.00 2.16 2.31 3.18 2.11 1.15 0.46 2.16 1.18 3.22 2.24 1.23 1.22 2.76 2.49 1.11 3.31 3.34

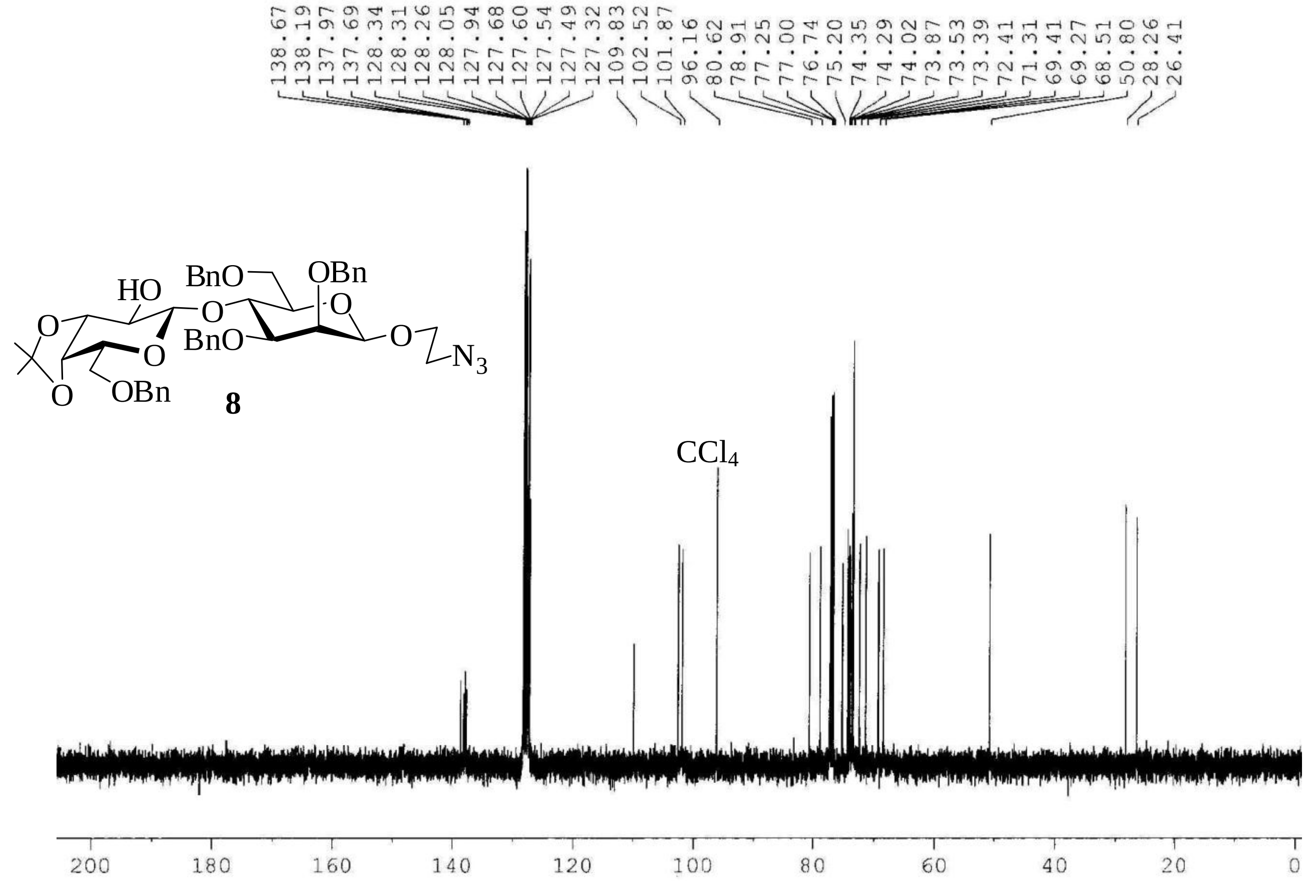
138.67 138.19 137.97 137.69 128.34 128.31 128.26 128.05 127.94 127.68 127.60 127.54 127.49 127.32 109.83 102.52 101.87 96.16 80.62 78.91 77.25 77.00 76.74 75.20 74.35 74.29 74.02 73.87 73.53 73.39 72.41 71.31 69.41 69.27 68.51 50.80 28.26 26.41
HO
BnO
OBn
O
BnO
O
N_3
O
OBn
8
CCl_4
200 180 160 140 120 100 80 60 40 20 0

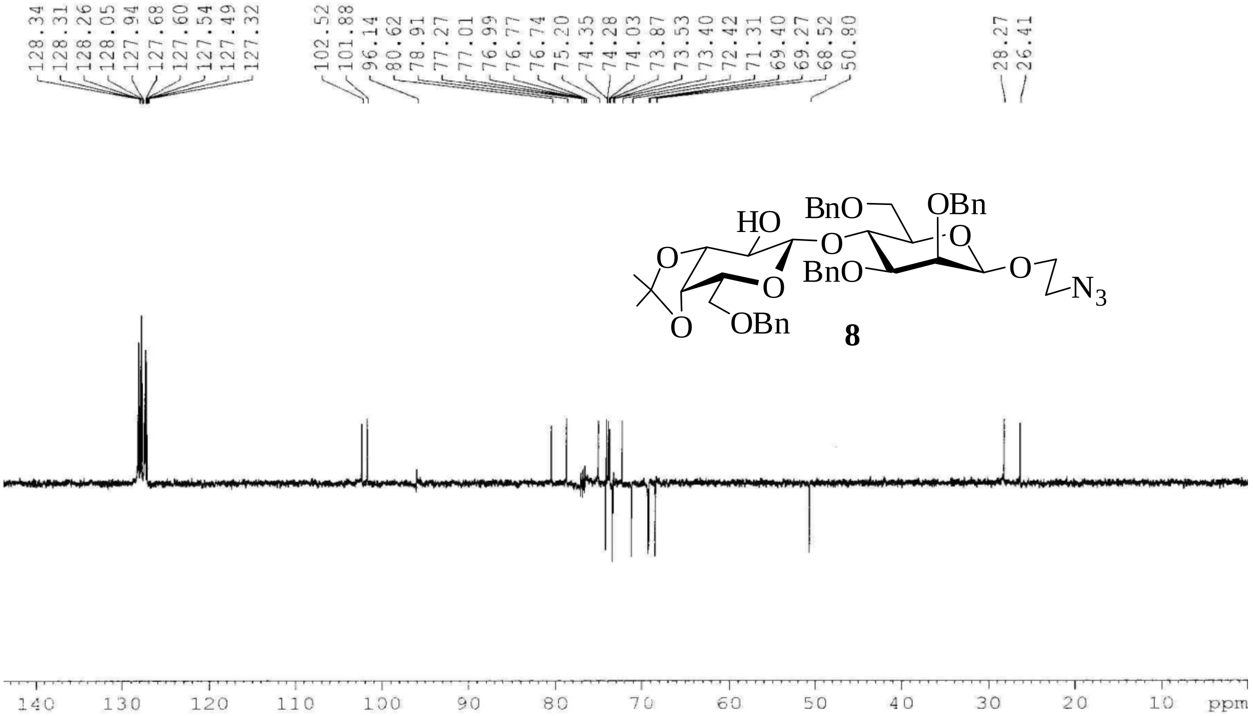
128.34
128.31
128.26
128.05
127.94
127.68
127.60
127.54
127.49
127.32
102.52
101.88
96.14
80.62
78.91
77.27
77.01
76.99
76.77
76.74
75.20
74.35
74.28
74.03
73.87
73.53
73.40
72.42
71.31
69.40
69.27
68.52
50.80
28.27
26.41
HO
BnO
OBn
O
O
O
BnO
O
O
N3
O
O
OBn
8
140 130 120 110 100 90 80 70 60 50 40 30 20 10 ppm

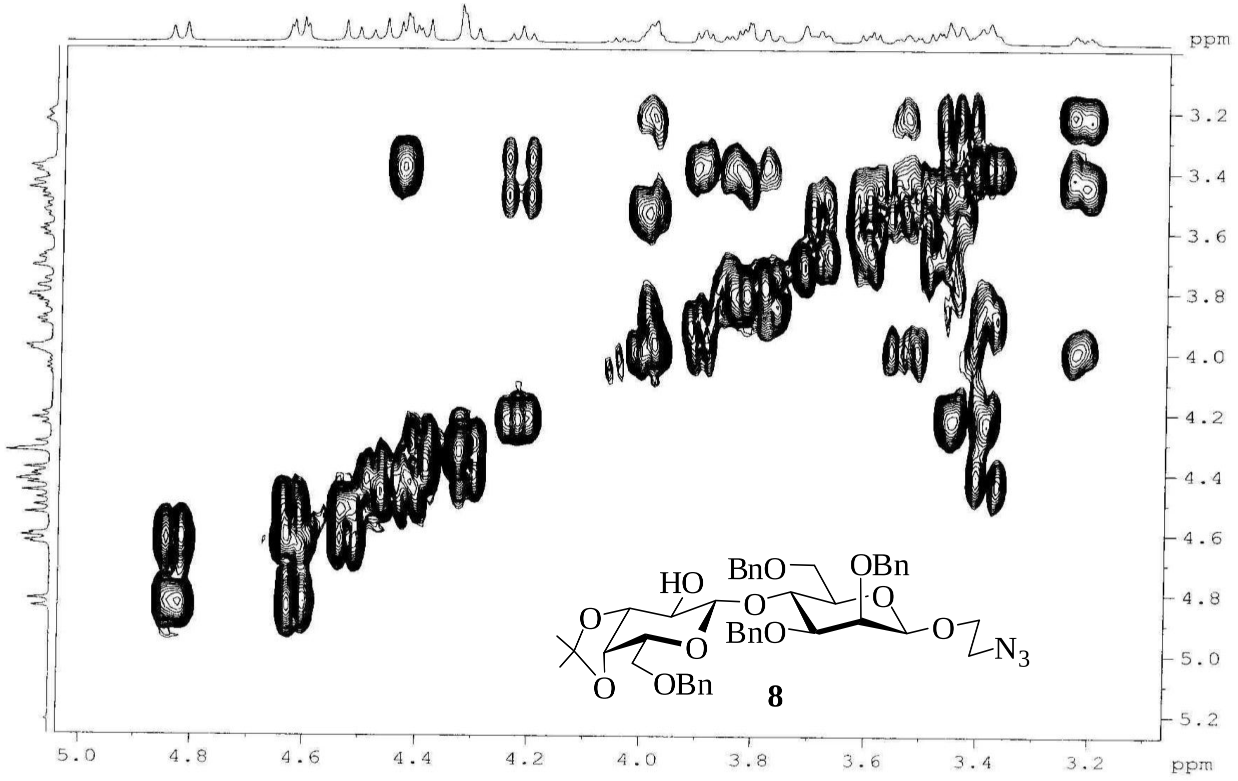
ppm
3.2
3.4
3.6
3.8
4.0
4.2
4.4
4.6
4.8
5.0
5.2
HO
BnO
OBn
O
O
O
BnO
O
O
N3
O
O
OBn
8
5.0 4.8 4.6 4.4 4.2 4.0 3.8 3.6 3.4 3.2 ppm

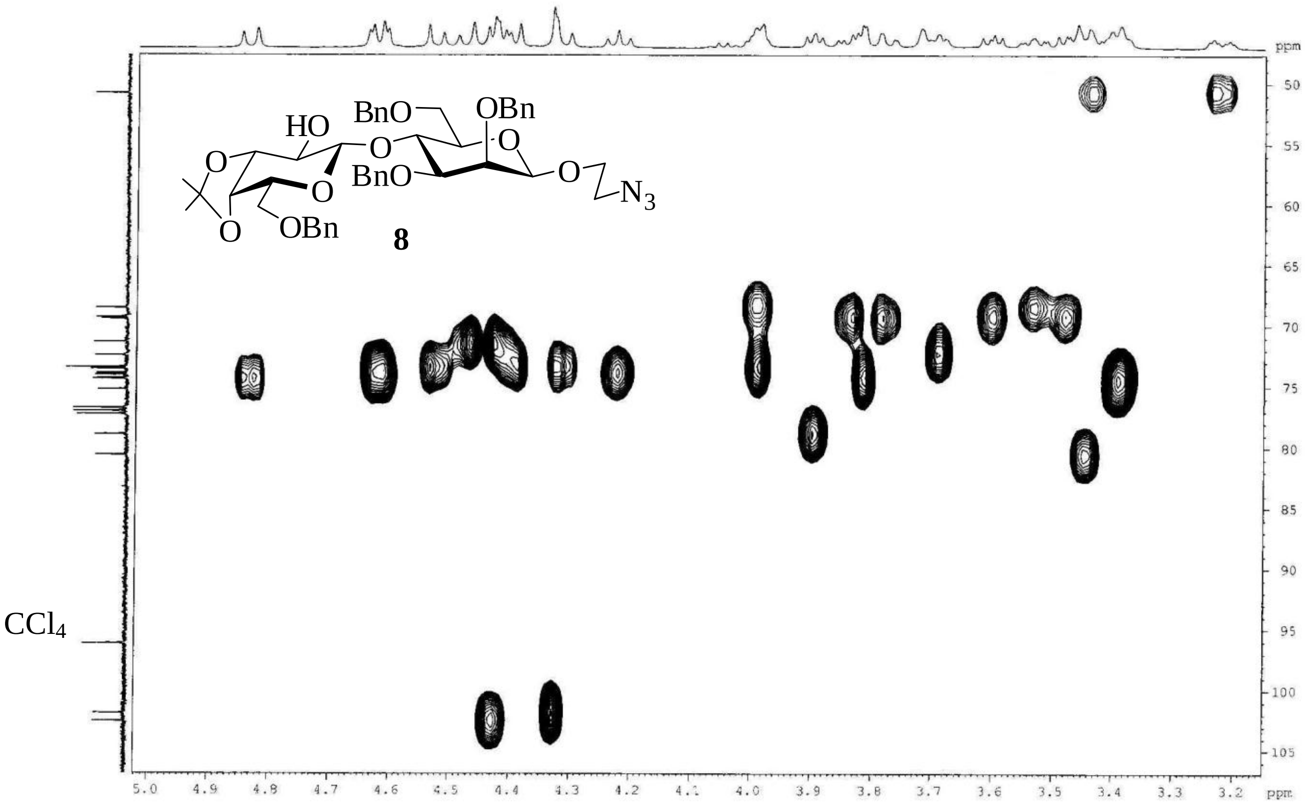

CCl4
8
ppm

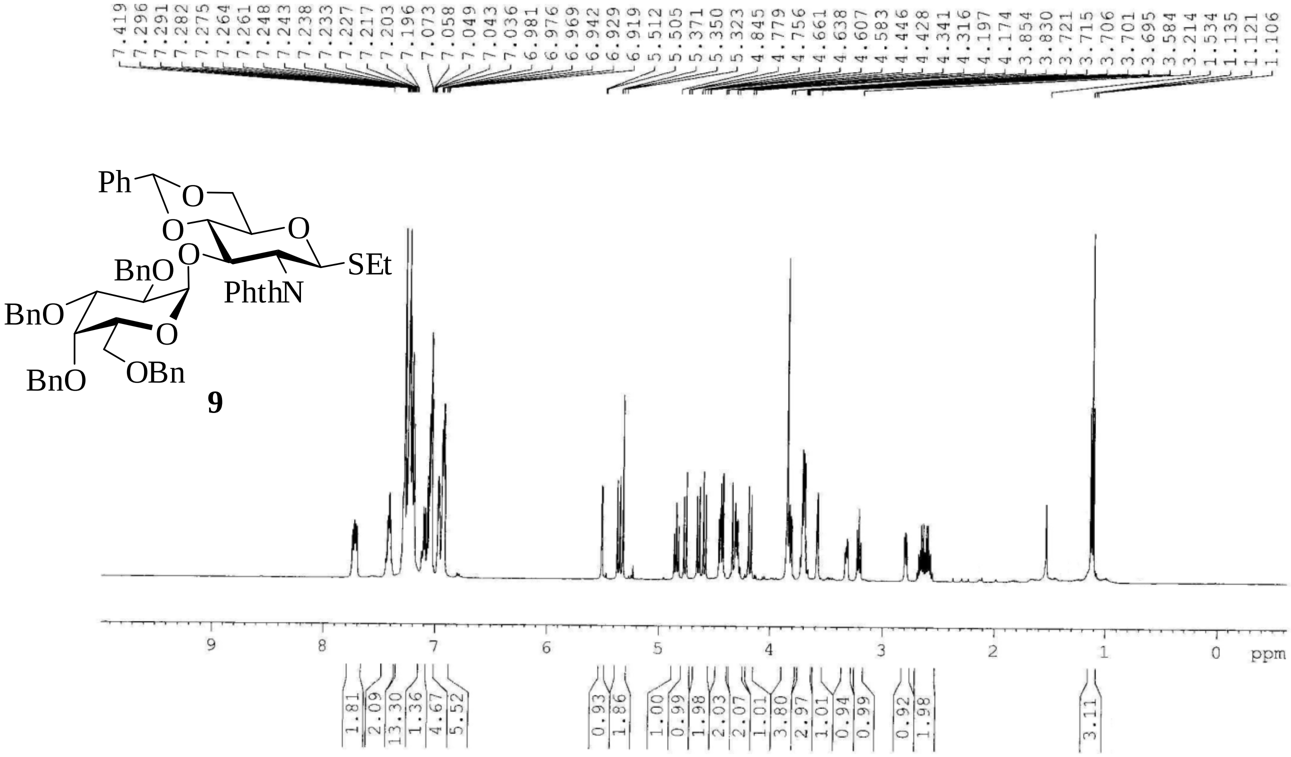
7.419
7.296
7.291
7.282
7.275
7.264
7.261
7.248
7.243
7.238
7.233
7.227
7.217
7.203
7.196
7.073
7.058
7.049
7.043
7.036
6.981
6.976
6.969
6.942
6.929
6.919
5.512
5.505
5.371
5.350
5.323
4.845
4.779
4.756
4.661
4.638
4.607
4.583
4.446
4.428
4.341
4.316
4.197
4.174
3.854
3.830
3.721
3.715
3.706
3.701
3.695
3.584
3.214
1.534
1.135
1.121
1.106
9 8 7 6 5 4 3 2 1 0 ppm
1.81
2.09
13.30
1.36
4.67
5.52
0.93
1.86
1.00
0.99
1.98
2.03
2.07
1.01
3.80
2.97
1.01
0.94
0.99
0.92
1.98
3.11
Ph
O
O
O
O
BnO
SEt
PhthN
BnO
O
BnO
OBn

9

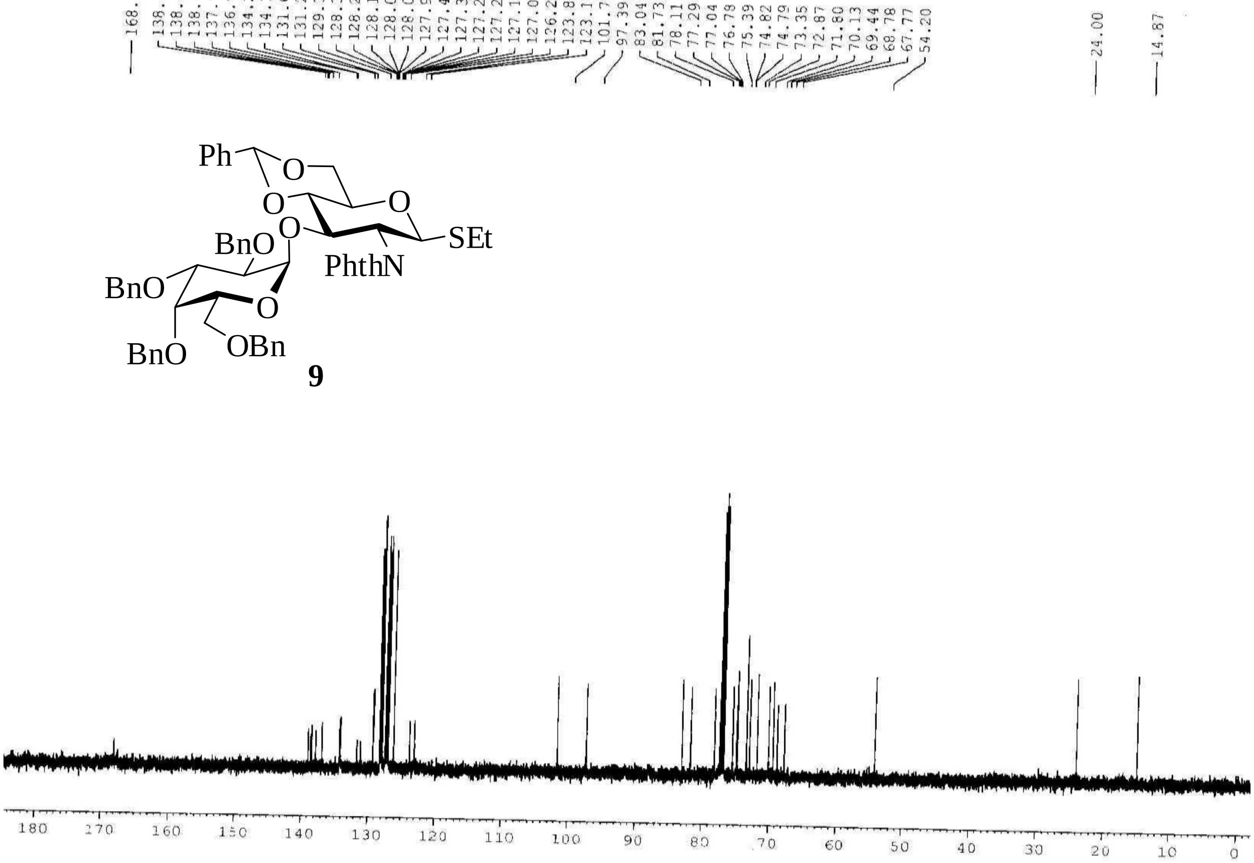
168.08
138.93
138.59
138.43
137.92
136.93
134.27
134.18
131.68
131.23
129.32
128.37
128.24
128.14
128.03
128.02
127.97
127.44
127.30
127.27
127.21
127.11
127.09
126.27
123.89
123.11
101.70
97.39
83.04
81.73
78.11
77.29
77.04
76.79
75.39
74.82
74.79
73.35
72.87
71.80
70.13
69.44
68.78
67.77
54.20
24.00
14.87
180 170 160 150 140 130 120 110 100 90 80 70 60 50 40 30 20 10 0
Ph
O
O
O
O
BnO
SEt
PhthN
BnO
O
BnO
OBn

9

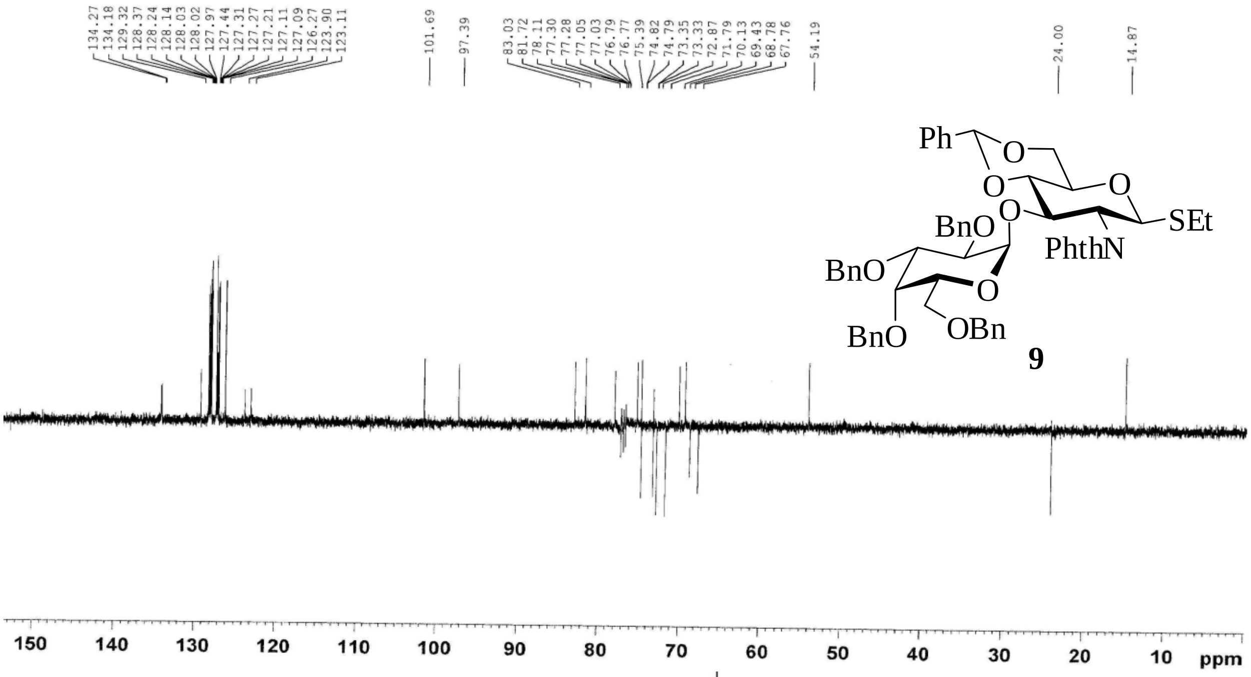
134.27
134.18
129.32
128.37
128.24
128.14
128.03
128.02
127.97
127.44
127.31
127.27
127.21
127.11
127.09
126.27
123.90
123.11
101.69
97.39
83.03
81.72
78.11
77.30
77.28
77.05
77.03
76.79
76.77
75.39
74.82
74.79
73.35
73.33
72.87
71.79
70.13
69.43
68.78
67.76
54.19
24.00
14.87
Ph
O
O
O
O
BnO
SEt
PhthN
BnO
O
BnO
OBn
9
150 140 130 120 110 100 90 80 70 60 50 40 30 20 10 ppm

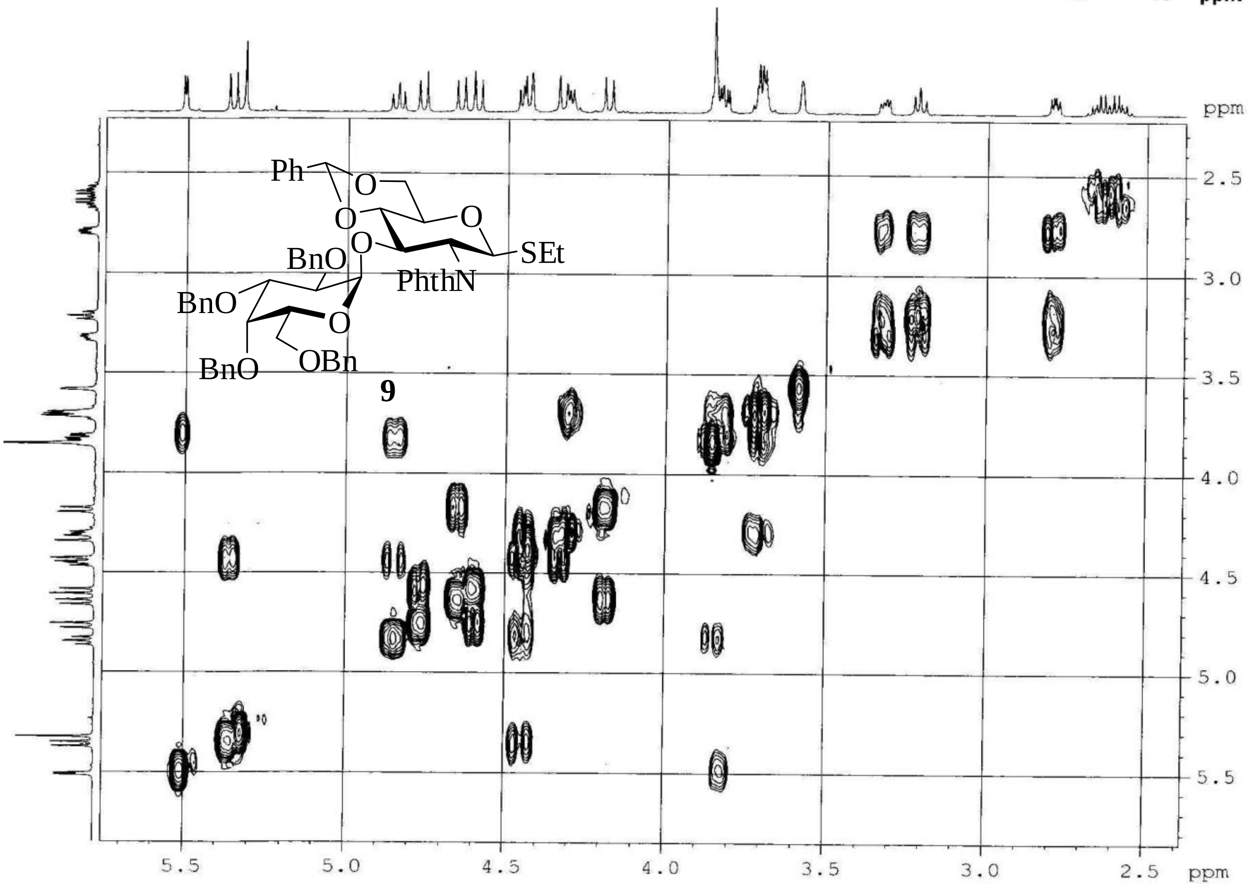
ppm
Ph
O
O
O
O
BnO
SEt
PhthN
BnO
O
BnO
OBn
9
2.5
3.0
3.5
4.0
4.5
5.0
5.5
5.5 5.0 4.5 4.0 3.5 3.0 2.5 ppm

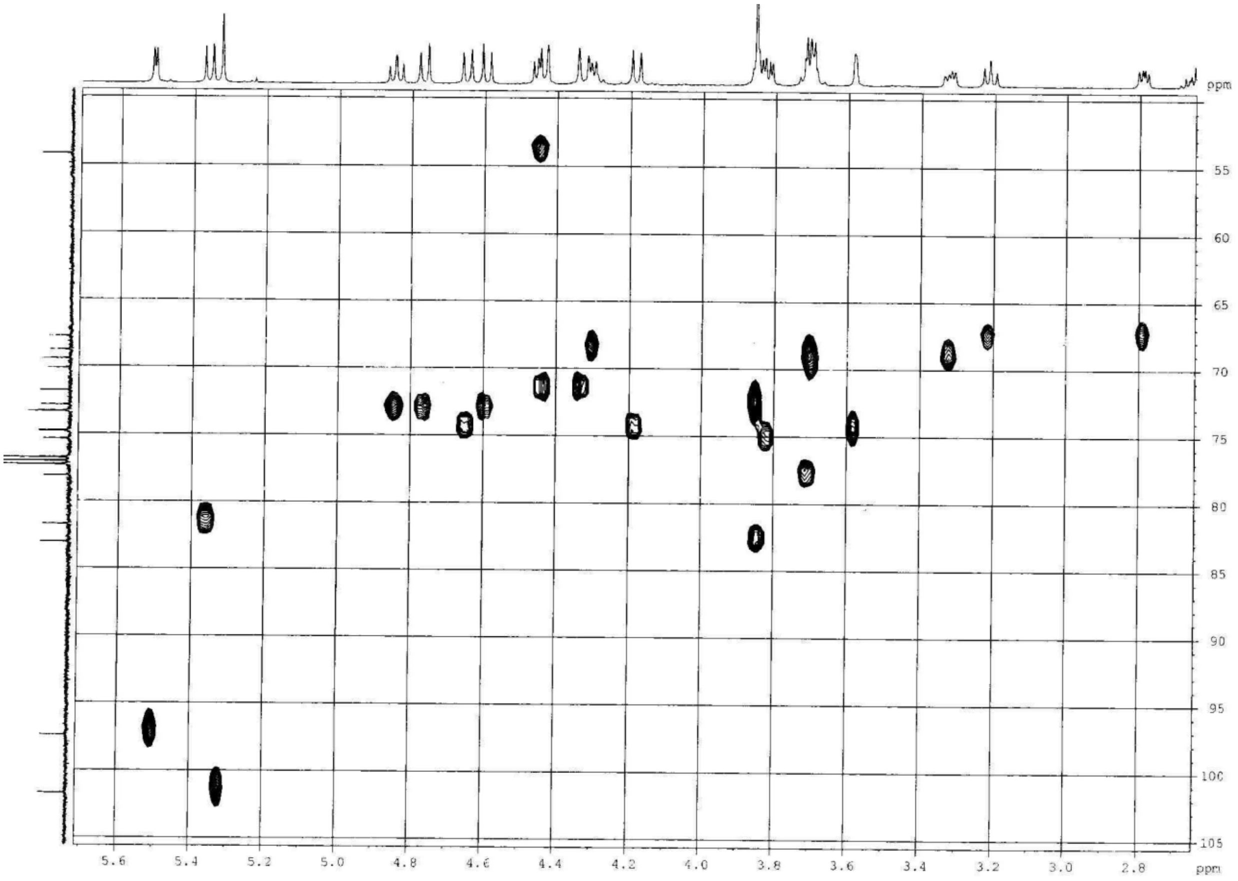

ppm
55
60
65
70
75
80
85
90
95
100
105
5.6
5.4
5.2
5.0
4.8
4.6
4.4
4.2
4.0
3.8
3.6
3.4
3.2
3.0
2.8
ppm

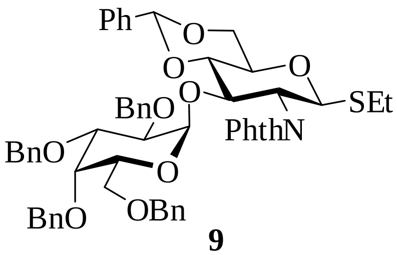

Ph
O
O
O
O
SEt
BnO
PhthN
BnO
O
BnO
OBn

9

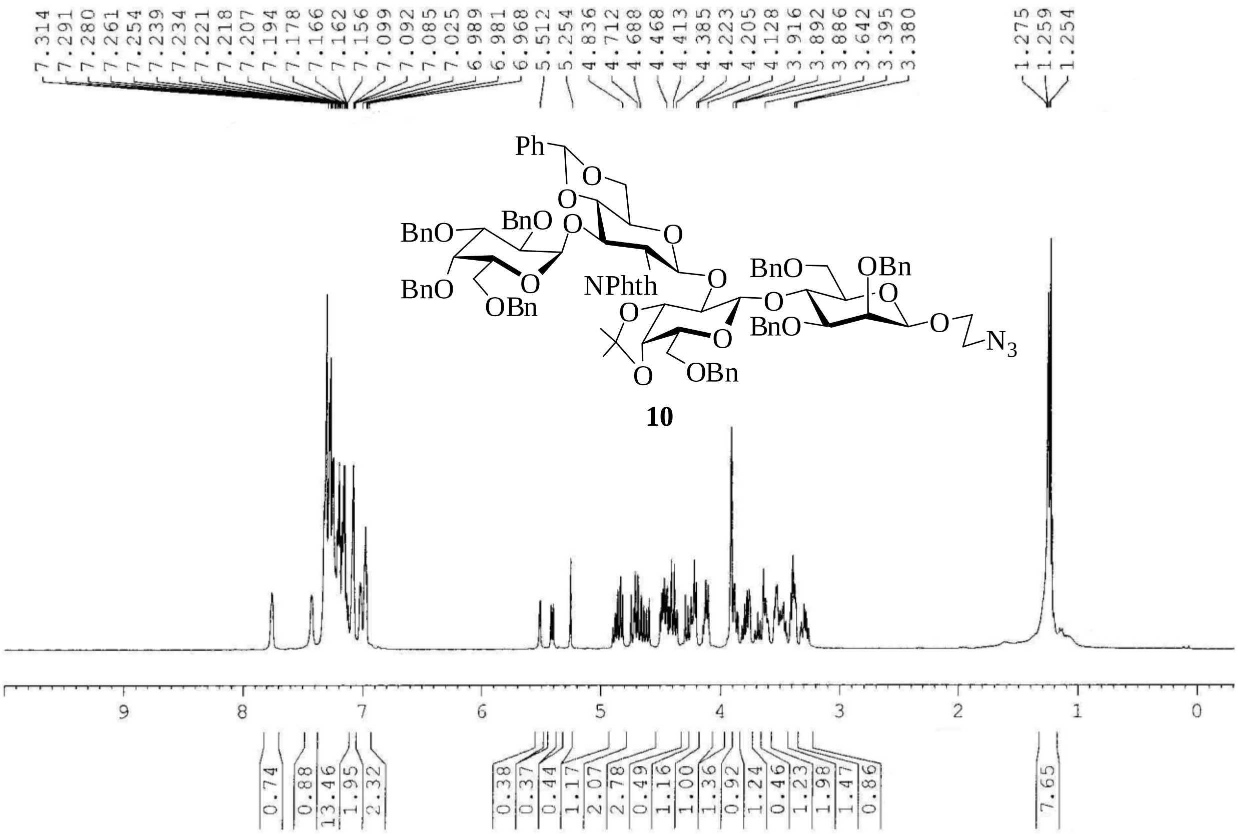
7.314 7.291 7.280 7.261 7.254 7.239 7.234 7.221 7.218 7.207 7.194 7.178 7.166 7.162 7.156 7.099 7.092 7.085 7.025 6.989 6.981 6.968 5.512 5.254 4.836 4.712 4.688 4.468 4.413 4.385 4.223 4.205 4.128 3.916 3.892 3.886 3.642 3.395 3.380 1.275 1.259 1.254
Ph
BnO
BnO
NPhth
OBn
OBn
BnO
BnO
N3
10
9 8 7 6 5 4 3 2 1 0
0.74 0.88 13.46 1.95 2.32 0.38 0.37 0.44 1.17 2.07 2.78 0.49 1.16 1.00 1.36 0.92 1.24 0.46 1.23 1.98 1.47 0.86 7.65

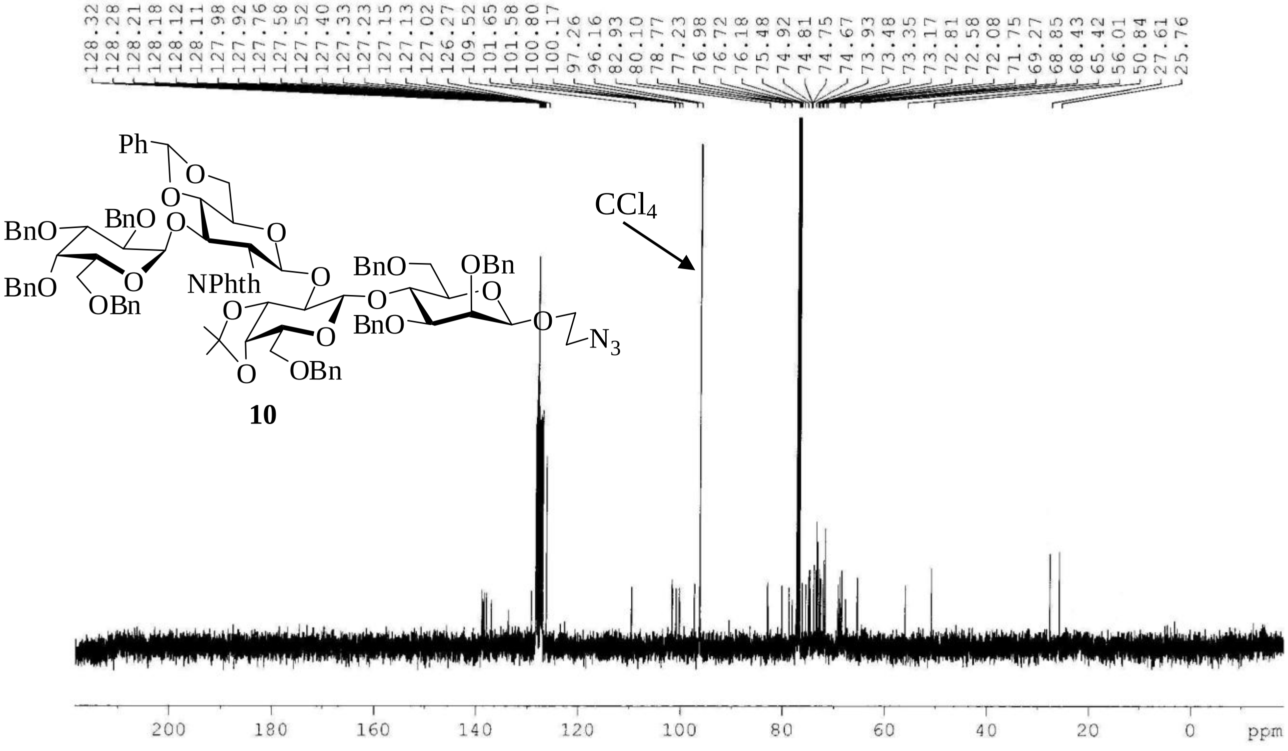
128.32 128.28 128.21 128.18 128.12 128.11 127.98 127.92 127.76 127.58 127.52 127.40 127.33 127.23 127.15 127.13 127.02 126.27 109.52 101.65 101.58 100.80 100.17 97.26 96.16 82.93 80.10 78.77 77.23 76.98 76.72 76.18 75.48 74.92 74.81 74.75 74.67 73.93 73.48 73.35 73.17 72.81 72.58 72.08 71.75 69.27 68.85 68.43 65.42 56.01 50.84 27.61 25.76
Ph
BnO
BnO
NPhth
OBn
OBn
BnO
BnO
N3
10
CCl4
200 180 160 140 120 100 80 60 40 20 0 ppm

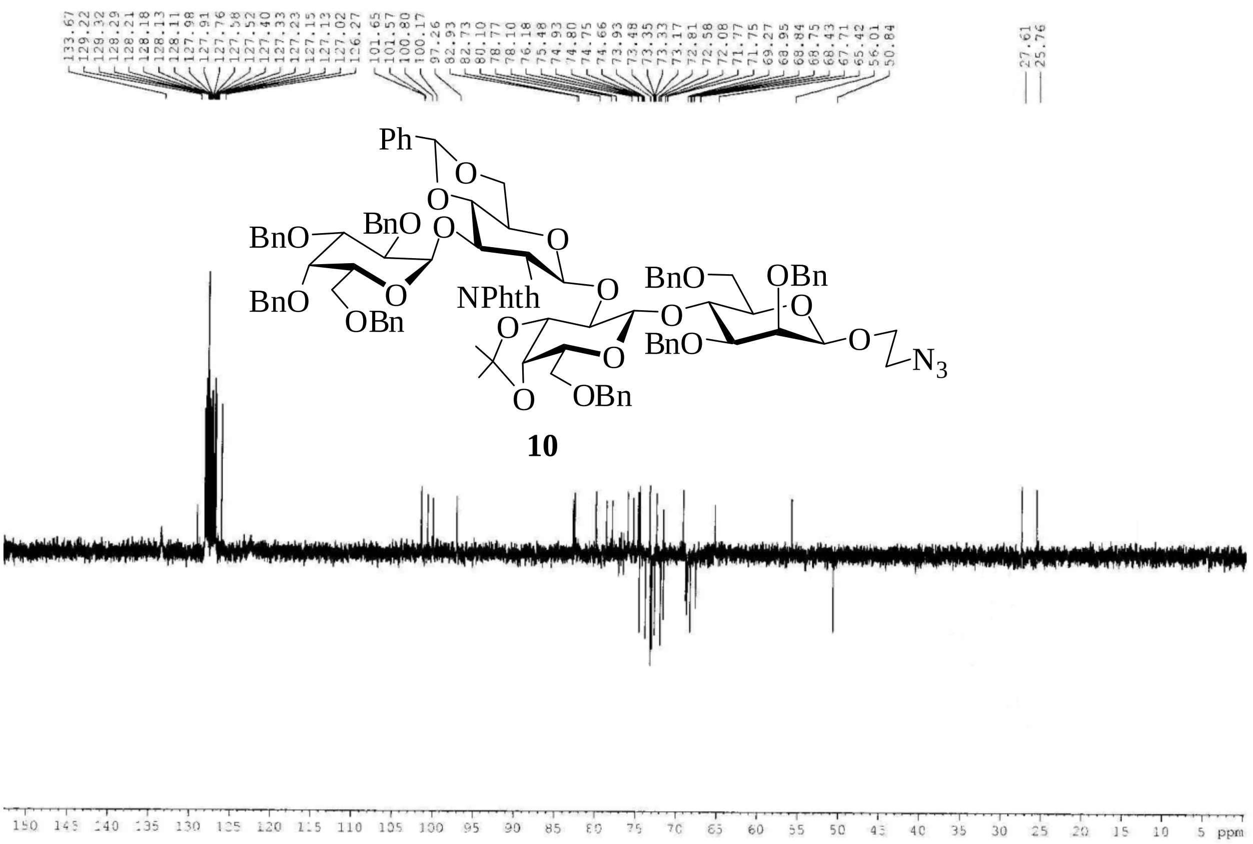

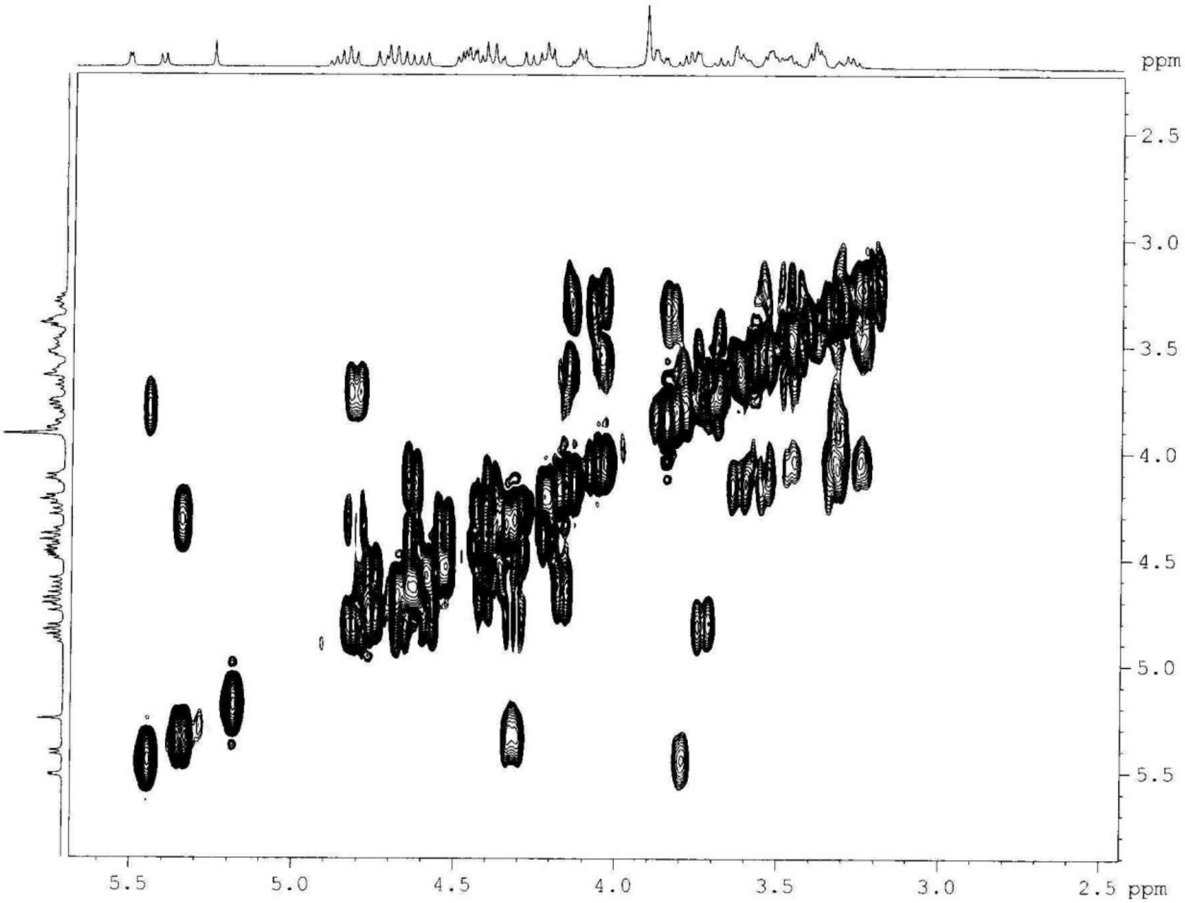
ppm
2.5
3.0
3.5
4.0
4.5
5.0
5.5
5.5
5.0
4.5
4.0
3.5
3.0
2.5 ppm

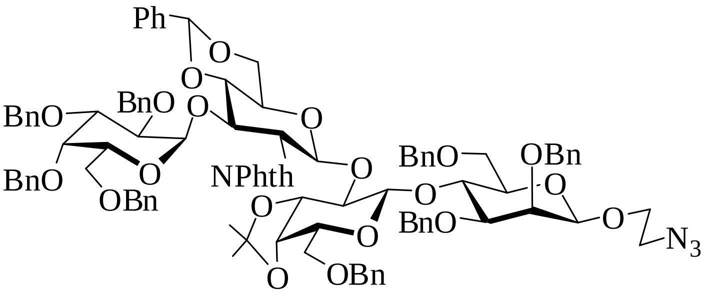
Ph
O
O
BnO
BnO
O
O
BnO
O
NPhth
BnO
OBn
OBn
O
O
BnO
O
O
O
N3
O
O
OBn

10

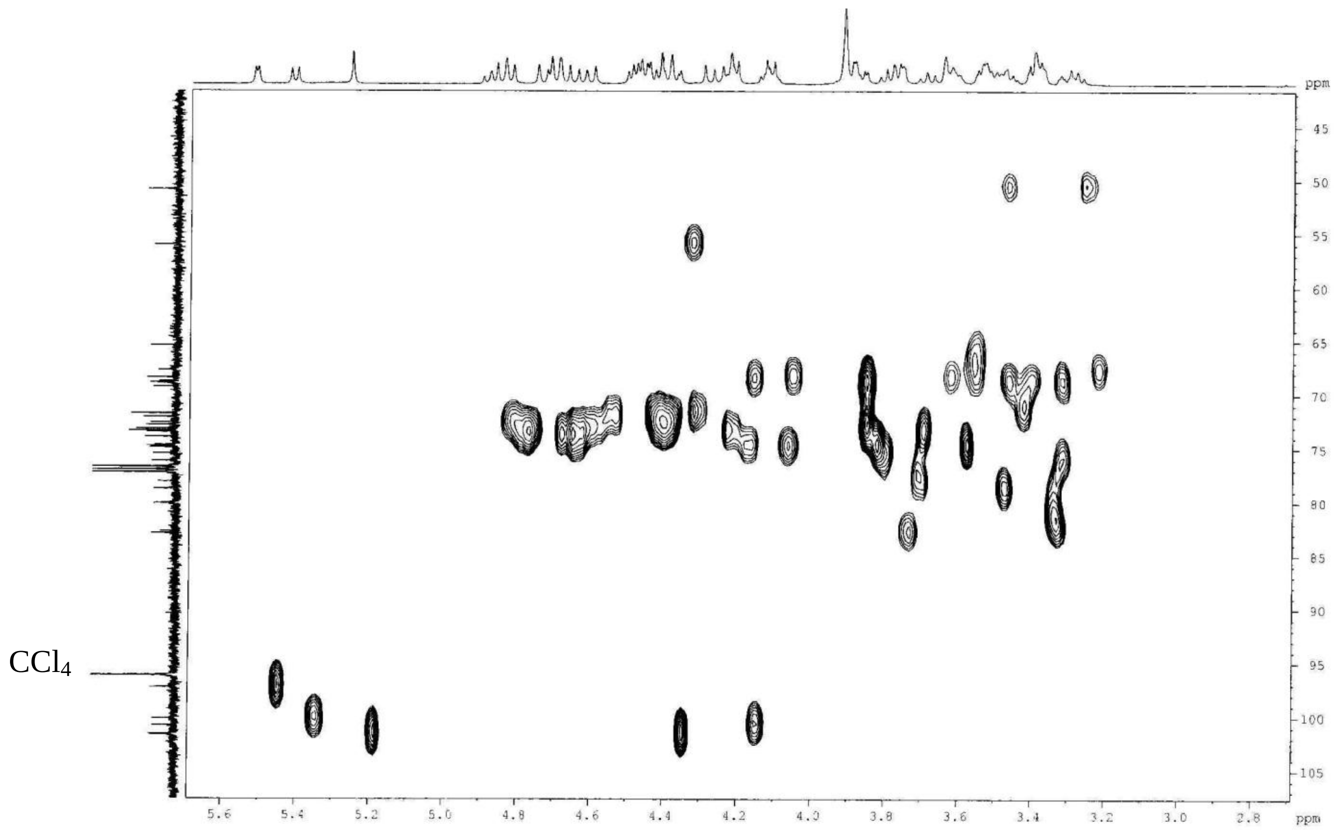
CCl4
ppm
45
50
55
60
65
70
75
80
85
90
95
100
105
5.6
5.4
5.2
5.0
4.8
4.6
4.4
4.2
4.0
3.8
3.6
3.4
3.2
3.0
2.8

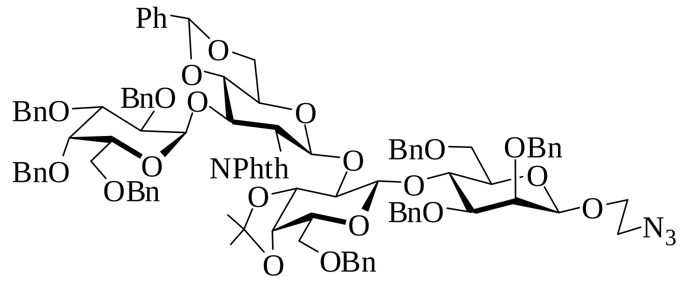
Ph
O
O
BnO
BnO
O
O
BnO
O
NPhth
O
BnO
OBn
OBn
O
O
O
O
BnO
O
O
N3
O
OBn

10
